# Aβ-induced mitochondrial dysfunction in neural progenitors controls KDM5A to influence neuronal differentiation

**DOI:** 10.1038/s12276-022-00841-w

**Published:** 2022-09-02

**Authors:** Dong Kyu Kim, Hyobin Jeong, Jingi Bae, Moon-Yong Cha, Moonkyung Kang, Dongjin Shin, Shinwon Ha, Seung Jae Hyeon, Hokeun Kim, Kyujin Suh, Mi-Sun Choi, Hoon Ryu, Seong-Woon Yu, Jong-Il Kim, Yeon-Soo Kim, Sang-Won Lee, Daehee Hwang, Inhee Mook-Jung

**Affiliations:** 1 Department of Biomedical Science, College of Medicine, Seoul National University, Seoul, Korea; 2 Dementia Research Center, Seoul National University College of Medicine, Seoul, Korea; 3 European Molecular Biology Laboratory, Genome Biology Unit, Heidelberg, Germany; 4 Department of Biological Sciences, Seoul National University, Seoul, Korea; 5 Department of Chemistry, Center for Proteogenome Research, Korea University, Seoul, Korea; 6 LG Chem Life Science R&D Campus, Drug Discovery Center, Seoul, Korea; 7 Graduate School of New Drug Discovery & Development, Chungnam National University, Daejeon, Korea; 8 Department of Brain and Cognitive Sciences, Daegu Gyeongbuk Institute of Science and Technology, Daegu, Korea; 9 Center for Neuroscience, Brain Science Institute, Korea Institute of Science and Technology, Seoul, Korea; 10 Department of Predictive Toxicology, Korea Institute of Toxicology (KIT), Daejeon, Korea; 11 Medical Research Center, Genomic Medicine Institute (GMI), Seoul National University, Seoul, Korea

**Keywords:** Neural stem cells, Neural stem cells, Alzheimer's disease

## Abstract

Mitochondria in neural progenitors play a crucial role in adult hippocampal neurogenesis by being involved in fate decisions for differentiation. However, the molecular mechanisms by which mitochondria are related to the genetic regulation of neuronal differentiation in neural progenitors are poorly understood. Here, we show that mitochondrial dysfunction induced by amyloid-beta (Aβ) in neural progenitors inhibits neuronal differentiation but has no effect on the neural progenitor stage. In line with the phenotypes shown in Alzheimer’s disease (AD) model mice, Aβ-induced mitochondrial damage in neural progenitors results in deficits in adult hippocampal neurogenesis and cognitive function. Based on hippocampal proteome changes after mitochondrial damage in neural progenitors identified through proteomic analysis, we found that lysine demethylase 5A (KDM5A) in neural progenitors epigenetically suppresses differentiation in response to mitochondrial damage. Mitochondrial damage characteristically causes KDM5A degradation in neural progenitors. Since KDM5A also binds to and activates neuronal genes involved in the early stage of differentiation, functional inhibition of KDM5A consequently inhibits adult hippocampal neurogenesis. We suggest that mitochondria in neural progenitors serve as the checkpoint for neuronal differentiation via KDM5A. Our findings not only reveal a cell-type-specific role of mitochondria but also suggest a new role of KDM5A in neural progenitors as a mediator of retrograde signaling from mitochondria to the nucleus, reflecting the mitochondrial status.

## Alzheimer’s disease: a molecular link with mitochondrial damage

Neural Stem cells in the brain that normally generate new neurons throughout life can become dysfunctional due to damage caused by the protein amyloid-beta to mitochondria, the intracellular energy-producing structures. Neuron generation is reduced in Alzheimer’s disease, therefore understanding its causes could lead to new treatments. Dong Kyu Kim at Seoul National University, South Korea, and colleagues studied neuron generation in the hippocampus region of mouse brains. They found that the protein amyloid-beta, implicated in causing Alzheimer’s, can induce dysfunctional neuron generation by damaging mitochondria in a way that affects the activity of a gene-regulating protein called KDM5A. The research reveals a central role for mitochondria of neural stem cells in neuron generation, and suggests that drugs supporting mitochondrial function might offer a new approach to treat Alzheimer’s and perhaps other degenerative brain diseases.

## Introduction

Adult hippocampal neurogenesis (AHN) is the process by which neural stem cells continue to divide and form new neurons even after birth^1^. Adult neurogenesis occurs in the subventricular zone (SVZ) of the lateral ventricle and the subgranular zone (SGZ) of the hippocampus^2^. Neural stem cells (NSCs) in the SVZ and the SGZ pass through different stages as they differentiate into mature neurons, joining existing neuronal circuits and contributing to memory and cognitive functions^3^. During AHN, diverse transcription factors and epigenetic regulators cooperatively activate the sequential transcription required at each step. Unique marker proteins transcribed at each step are known for each cell type, enabling researchers to quantify AHN by investigating the transient expression patterns of marker proteins^4^.

The hippocampus is vulnerable to Alzheimer’s disease (AD). A recent study showed that AHN continues to occur even in old age but is significantly reduced in the hippocampus of AD patients^5^. Other studies have shown that alterations in AHN are related to pathological features in AD brains and AD model mice, including amyloid pathology in the hippocampus^6–8^. In AD patient brains, the number of immature neurons—doublecortin^+^ (DCX^+^) cells—in the SGZ drastically decreased beginning at Braak stage 1. However, the number of DCX^+^ cells that expressed calretinin, which is transiently expressed in immature neurons at the early differentiation phase, did not change significantly according to Braak stage, indicating that AD pathology has different effects on each stage of AHN and dysregulates neuronal differentiation rather than affecting neural stem/progenitor cells^5^. However, relatively little work has focused on studying the molecular mechanisms underlying the dysregulation of AHN by AD pathology.

Stem cells depend on glycolytic metabolism rather than mitochondrial oxidative phosphorylation (OXPHOS)^9^. Emerging evidence indicates that in addition to controlling energy production, mitochondria centrally coordinate NSC fate decisions during AHN by managing metabolites, redox signaling, and the epigenetic state of the NSC^10^. Mitochondrial dynamics in neural stem cells also affects fate plasticity in the postmitotic period during neurogenesis^11^. Abnormal mitochondrial activity impairs the maintenance of stem cells and their fate decisions^12,13^. Disruption of mitochondrial metabolism in NSCs perturbs the progression of AHN, indicating that there is a close relationship between NSC metabolism and differentiation^14^.

Mitochondrial retrograde signaling is a pathway that modulates transcription to maintain cellular homeostasis through retrograde signals originating from mitochondria^15^. Several mediators, such as mitochondria-derived peptides, mitochondrial DNA, and metabolites, can transmit mitochondrial signals to the nucleus in response to metabolic stress, which in turn regulates transcription^16,17^. However, the mode of retrograde signaling in which epigenetic regulators required for neuronal differentiation can be regulated by mitochondria has not been elucidated. The stability and turnover of epigenetic regulators could affect the transcriptional activity influencing the fate decisions of stem cells, which deteriorates during senescence and aging^18^. Histone-modifying enzymes are controlled by the ubiquitin‒proteasome system in response to various stimuli, and this regulation is critical for regulating chromatin structure and gene expression^19^. Here, we show that mitochondrial dysfunction of neural progenitors induced by Aβ inhibits neuronal differentiation. Additionally, we find that mitochondrial damage affects the proteostasis of the epigenetic regulator lysine demethylase 5A (KDM5A), resulting in dysregulation of AHN and cognitive decline. Given that mitochondrial dysfunction is a characteristic feature of the AD brain, mitochondrial damage caused by Aβ is sufficient to induce AHN deficits in AD. Collectively, our results emphasize that mitonuclear communication through KDM5A in neural progenitors functions as a regulatory hub of AHN and that restoration of mitochondria in neural progenitors should be considered a potential therapeutic target in efforts to ameliorate AHN defects in AD.

## Materials and methods

The methodologies, including mitochondrial fractionation, transmission electron microscopy imaging, chromatin immunoprecipitation, and proteomic experiments, are described in detail in the Supplementary Information.

### Animals

5XFAD mice (Tg6799; The Jackson Laboratory, Stock #006554), which express mutant human amyloid precursor protein (APP) with the Swedish mutation (K670N, M671L), the Florida mutation (I716V), and the London mutation (V717I) as well as mutant human presenilin1 (PS1) with the M146L and L286V mutations under transcriptional control of the mouse Thy-1 promoter, were used. All experiments were performed using female 5XFAD mice. In addition, 8-week-old C57BL6 male mice were used for virus injection. All animal experiments and management procedures were performed as outlined in the guidelines of the Institutional Animal Care and Use Committee of Seoul National University.

### Real-time qPCR

Total RNA was isolated by using an RNeasy Plus Mini Kit (QIAGEN). cDNA was synthesized from 100 to 200 ng/μl of total RNA by a Maxime RT PreMix Kit (iNtRON Biotechnology). The mRNA levels of genes of interest were measured by real-time qPCR with a KAPA SYBR FAST ABI Prism qPCR Kit (KAPA Systems) and normalized to the level of 18S ribosomal RNA (18S rRNA) as a housekeeping gene. The PCR primers used in the study are listed in Supplementary Table 1.

### Real-time measurement of the oxygen consumption rate

The oxygen consumption rate (OCR) was measured by using a Seahorse XF24 analyzer (Seahorse Bioscience). A total of 4 × 10^4^ cells were plated in XF24 cell culture microplates and cultured. The cartridge plate was incubated with XF Calibrant buffer for one day (37 °C, CO_2_-free); analytical medium (XF basal medium supplemented with 1 mM pyruvate, 4 mM glutamine, and 25 mM glucose) was prepared immediately before analysis.

### Immunohistochemistry

Mice were anesthetized and perfused with cold PBS. The whole brain was fixed with 4% paraformaldehyde solution for 24 h at 4 °C. Next, the fixed brain was incubated in 30% sucrose solution for 72 h at 4 °C. Brain tissue was cut to a 30-μm thickness using a cryostat (Leica). Brain sections were washed in PBS and then incubated with 70% formic acid solution for antigen retrieval. The sections were immersed in a solution with BSA, 0.3% Triton X-100, and 5% horse serum overnight at room temperature to block nonspecific binding and enhance antibody permeability. Next, the sections were incubated with primary antibodies against the target proteins overnight at 4 °C. The primary antibodies included anti-Iba-1 (Wako; 1:1000), anti-GFAP (Invitrogen, 1:1000), anti-SOX2 (Abcam, 1:100), anti-Calbindin (Cell Signaling, 1:200), anti-4G8 (Biolegend, 1:500), anti-DCX (Santa Cruz, 1:200), and anti-synaptoporin (SYSY, 1:250). After reaction with primary antibodies, the sections were incubated with secondary antibodies (Invitrogen; 1:400) for 1 h at room temperature. Finally, the sections were incubated with DAPI (0.4 μg/ml) to stain nuclei. Mounted brain sections were imaged by an LSM700 confocal laser scanning microscope (Carl Zeiss), and images were analyzed by ImageJ software.

### Behavioral tests

1. Y-maze test
  Mice were subjected to spatial adaptation and habituation in a Y-shaped maze one day before the test day. On the following day, the mice were allowed to freely explore the Y-maze for 8 min. The results for spatial memory were analyzed by calculating the percentage of spontaneous alternations made in the three different arms and the total number of entrances into the maze.
2. Novel object recognition (NOR) test
  Mice injected with LV-scramble siRNA or LV-*Kdm5a* siRNA were habituated to an empty rectangular box for 10 min on day 1. On day 2, two identical objects were placed at the corner of the box, 5 cm away from the same wall, and then individual mice were allowed to explore freely in the box for 10 min. After 4 h, one familiar object was replaced with a novel object, and we then measured the time spent exploring each object over an 8 min period. Recognition memory was assessed by the discrimination index, which was calculated as the percentage of the total time spent exploring a novel object during the test session.
3. Passive avoidance task (PAT)
  Mice injected with LV-GFP or LV-mitoAβ were allowed to explore a chamber divided into a lighted and a dark compartment by a gate. Each mouse experienced an electric shock (0.7 mA, 3 s) only in the dark compartment in the acquisition trial. On the following day, the mice were placed back in the chamber and allowed to explore the chamber for 300 s. Learning and memory were evaluated by measuring the latency time to cross between the compartments.

### Mitochondrial ROS measurement

Mitochondria-derived reactive oxygen species (ROS) were visualized using MitoSOX Red Mitochondrial Superoxide Indicator (M36008, Invitrogen). The dye was mixed in the cell culture medium at a final concentration of 5 μM. The reaction was carried out at 37 °C for 10 min, and the cells were then washed with PBS. Fluorescence signals were imaged using fluorescence microscopy (EVOS FL Auto2, Invitrogen).

### Mitochondrial membrane potential measurement

To visualize the mitochondrial membrane potential in neural progenitors, we treated cells with tetramethylrhodamine methyl ester perchlorate (TMRM; Invitrogen, T-668) at a final concentration of 100 nM in cell culture medium. After incubation for 30 min at 37 °C, stained cells were imaged using a live-cell imaging system (Evos FL Auto2, Invitrogen).

### Immunocytochemistry

Cells were washed with PBS and fixed with 4% PFA solution for 15 min at room temperature. After washing the cells with PBS, the samples were immersed in blocking/permeabilization solution containing BSA, 0.3% Triton X-100, and 5% horse serum for 1 h to improve the accessibility of the antibody. Next, the samples were incubated with primary antibodies diluted in blocking/permeabilization solution at 4 °C overnight. The primary antibodies included anti-KDM5A (Abcam, 1:100), anti-GFP (Invitrogen, 1:100), and anti-MAP2 (Abcam, 1:2000) antibodies. After staining with primary antibodies, samples were washed in PBS and incubated with secondary antibodies (Invitrogen, 1:400) for 1 h at room temperature. Finally, nuclei were stained with DAPI for 10 min at room temperature. Immunostained cells were mounted on glass slides and imaged using a ZEISS confocal microscope (LSM700). Images were analyzed by ImageJ software.

### Stereotaxic injection

Eight-week-old C57BL/6 male mice were anesthetized with zolazepam and tiletamine (30 mg/kg; Virbac), mixed with xylazine (10 mg/ml; Bayer Korea), and then fixed in a stereotaxic instrument (Neurostar). The coordinates for the injection were set as follows with respect to the bregma: AP axis, −2.0 mm; ML axis, 1.3 mm; and DV axis, 2.1 mm. A total volume of 2 μl (1 × 10^7^ lentiviral titer) was injected into the dorsal DG of the hippocampus using an automatic microinjector at a rate of 0.2 μl/min. After reaching the desired coordinates, a Hamilton syringe was left in place for 10 min to prevent backflow of the virus-containing liquid.

### Hippocampal neural stem cell culture

Hippocampal neural stem (HCN) cells isolated from adult rats were cultured at 37 °C and 5% CO_2_ on dishes coated with 10 μg/mL poly-l-ornithine (Sigma-Aldrich, P3655) and 5 μg/mL mouse laminin (Corning, 354232) and maintained in Dulbecco’s modified Eagle’s medium (DMEM)/F-12 (Invitrogen, 12400-024) containing 5 mg/L insulin (Sigma-Aldrich, I6634), 100 mg/L apo-transferrin (Prospec, PRO-325), penicillin (100 U/L)/streptomycin (100 mg/L) (HyClone, SV30010), 16 mg/L putrescin (Sigma-Aldrich, P5780), 30 nM sodium selenite (Sigma-Aldrich, S1382), 20 nM progesterone (Sigma-Aldrich, P0130), and 20 ng/ml basic fibroblast growth factor (bFGF) (Peprotech, #100-18B)^20^. For differentiation, HCN cells were plated into coated dishes at a density of ~0.5 × 10^5^ cells/cm^2^, and 24 h later, the culture medium was replaced with medium containing 1 µM retinoic acid (Enzo, BML-GR100), 5 µM forskolin (Enzo, BML-CN100), and 0.1% fetal bovine serum (Tissue Culture Biologicals, 101)^21^. Half of the medium was replaced every 2 days until the experiment.

### Analysis of upstream binding factors

We inferred upstream binding factors of proteins affected by LV-mitoAβ (281 upregulated DEPs and 218 downregulated DEPs) using ChIP-Atlas software^22^. We used the “Enrichment analysis” functionality of this software with the following parameters: (1) Genome: *M. musculus* (mm10), (2) Experiment type: ChIP TFs and others, (3) Cell-type Class: all cell types, (4) Search space: +−500 nt with respect to the transcription start sites (TSS). This analysis provided the *q* value as a significant measure of binding enrichment for each of the candidate regulators whose ChIP-seq profiles are available through this software. We selected potential candidate binding factors of upregulated and downregulated DEPs by extracting factors meeting the following two criteria: (1) *q* value < 0.0001 in ChIP-Atlas enrichment analysis and (2) identified in the LC‒MS/MS experiment in this study.

### Statistical analysis

All values are expressed as the means ± standard errors of the mean (SEMs). The significance of differences between two groups was analyzed by Student’s unpaired *t* test. For comparisons of three or more groups, the significance of differences was evaluated by one-way ANOVA or two-way ANOVA. Statistical significance is displayed as follows: N.S., not significant; *P < 0.05; **P < 0.01; ***P < 0.001; ****P < 0.0001.

## Results

### Mitochondrial dysfunction in neural progenitors inhibits neuronal differentiation

**Fig. 1:**
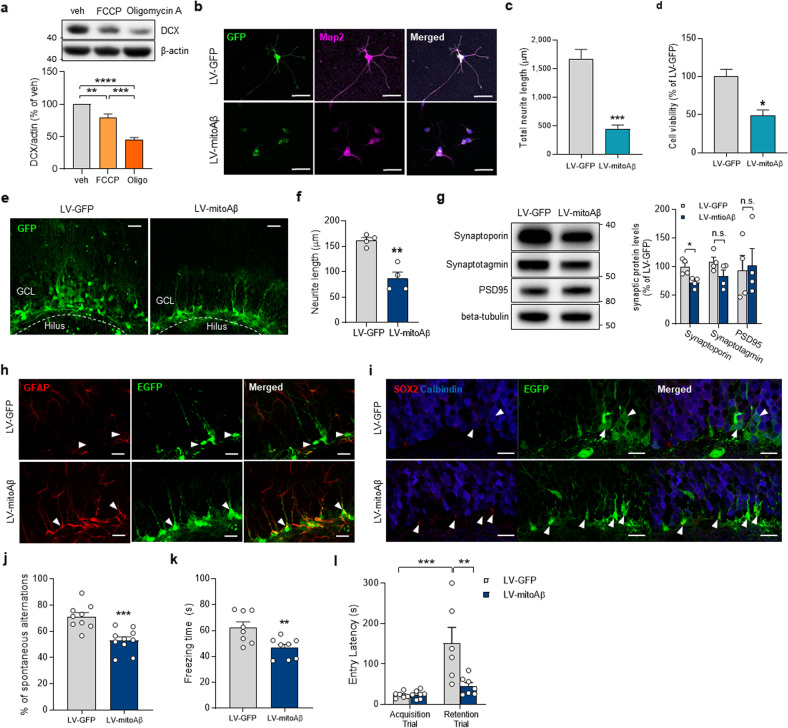
Mitochondrial dysfunction in neural stem/progenitors disrupts neural differentiation. **a** DCX protein levels in differentiated cells (12 days) after treatment of on neural progenitors with FCCP (3 μM, 12 h) or oligomycin A (3 μM, 12 h). One-way ANOVA, *n* = 4. **b** Representative images of neurons differentiated from hippocampal neural progenitors transduced with LV-GFP or LV-mitoAβ. Scale bar, 50 μm. **c** Quantitative analysis of total neurite length in differentiated neurons. Unpaired *t* test, *n* = 20 neurons in each group. **d** Quantitative analysis of viability in differentiated neurons. Unpaired *t* test, *n* = 3 in each group. **e** Representative images of neuronal differentiation in the SGZ after lentiviral transduction of GFP or mitoAβ for 8 weeks in vivo. GCL: Granule cell layer. Scale bar, 30 μm. **f** Quantitative analysis of neurite length in GFP/mitoAβ^+^ cells in the SGZ. Unpaired *t* test, *n* = 4 mice in each group. **g** Western blot analysis of synaptic proteins in the hippocampus of mice injected with LV-GFP and LV-mitoAβ. Data for each protein were normalized to the level of β-tubulin and the mean value in the LV-GFP group. Unpaired *t* test, *n* = 4 (LV-GFP) or 5 (LV-mitoAβ) mice. **h**, **i** Immunostaining for GFAP (neural stem cell marker), SOX2 (neural stem cell/progenitor/neuroblast marker), calbindin (differentiated neuron marker), and GFP (transduced cell marker) in the SGZ of mice injected with LV-GFP and LV-mitoAβ. Scale bar, 20 μm. **j** The percentage of spontaneous alternations in the Y-maze test. Unpaired *t* test, *n* = 9 (LV-GFP) or 10 (LV-mitoAβ) mice. **k** Freezing time in the CFC test. Unpaired t test, *n* = 8 in each group. **l** Entry latency in the PAT during the acquisition and retention trials. Two-way ANOVA, *n* = 6 (LV-GFP) or 7 (LV-mitoAβ) mice. All results are presented as the means ± SEMs. * *P* < 0.05; **P < 0.01; ***P < 0.001.

To verify whether mitochondrial dysfunction in neural progenitors affects neuronal differentiation, ReNcell CX immortalized human neural progenitor cells were differentiated after carbonyl cyanide-*p*-trifluoromethoxyphenylhydrazone (FCCP) or oligomycin A treatment at the progenitor stage, both of which lead to mitochondrial dysfunction by disrupting ATP synthesis across the mitochondrial inner membrane^23,24^. Differentiated cells treated with FCCP or oligomycin A at the progenitor stage expressed lower doublecortin (DCX) levels after 12 days of neuronal differentiation, indicating that the mitochondrial status in neural progenitors influences the differentiation process (Fig. 1a).

In the Alzheimer’s disease (AD) brain, amyloid-beta (Aβ) is found to accumulate in mitochondria, thereby causing mitochondrial dysfunction through binding to mitochondrial component proteins^25^. We previously reported a mitochondria-targeted Aβ (mitoAβ) construct that induces mitochondrial dysfunction, resulting in apoptotic cell death^26^. The MitoAβ construct is controlled by a dual promoter from which Aβ containing a signal peptide for mitochondrial translocation and eGFP, a marker for confirmation of expression, are coexpressed (Supplementary Fig. 1a). MitoAβ expression was induced by both transient transfection and viral transduction (Supplementary Fig. 1b, c), and the presence of mitoAβ in the mitochondria, not in the cytosol, was confirmed through mitochondrial enrichment fractionation (Supplementary Fig. 1c). To further investigate the structural and functional defects in mitochondria caused by mitoAβ, HT22 mouse hippocampal neuronal cells lentivirally transduced with GFP or GFP/mitoAβ (LV-GFP and LV-mitoAβ, respectively) were separated by fluorescence-activated cell sorting (FACS) to generate each homologous infected cell population (Supplementary Fig. 1d). Transmission electron microscopy imaging revealed aberrant mitochondrial cristae structures in mitoAβ-expressing cells (Supplementary Fig. 1e). We also examined functional abnormalities in mitochondria by measuring the cellular respiration rate with a Seahorse XF analyzer (Supplementary Fig. 1f). Our results confirmed that mitoAβ lowered the maximum respiration rate and the rate of mitochondrial ATP production but exerted no significant effect on the mitochondria-independent respiration rate (Supplementary Fig. 1g–i). These results indicate that mitoAβ causes structural and functional abnormalities in mitochondria.

Next, neural progenitors transduced with LV-GFP or LV-mitoAβ were differentiated to assess whether mitoAβ-induced mitochondrial damage in neural progenitors inhibits neuronal differentiation. GFP^+^ cells, as a control group, were well differentiated into neurons with normal neurite morphology; in contrast, GFP/mitoAβ^+^ cells had short and immature neurites, indicating their incomplete differentiation status (Fig. 1b, c). Likely as a result of incomplete differentiation, GFP/mitoAβ^+^ cells exhibited lower cell viability than control cells (Fig. 1d). These data suggest that mitochondrial dysfunction in neural progenitors dysregulates neuronal differentiation, supporting the idea that mitochondrial function is an essential checkpoint for the differentiation of neural progenitors.

### Mitochondrial dysfunction in the SGZ contributes to deficits in AHN

To further examine the effect of mitochondrial damage on AHN in vivo, we infused LV-GFP or LV-mitoAβ into the hippocampal SGZ of 8-week-old C576BL/6 mice. Two months after lentiviral transduction, the AHN status was analyzed, and behavioral tests were performed. In the control group, GFP^+^ cells extended long apical dendrites and migrated toward the upper granule cell layer in a normal neurogenesis process. In contrast, GFP/mitoAβ^+^ cells were confined to the SGZ and exhibited highly branched neurites similar to those of the radial glial-like stem cells observed in the early stage of AHN (Fig. 1e, f). We examined the hippocampal expression of several synaptic proteins, including synaptoporin, a granule cell-specific protein expressed in fully differentiated granule cells after the AHN process, to evaluate whether neural progenitors differentiated normally into mature neurons. There was no change in the expression of synaptotagmin and PSD95, but synaptoporin expression was significantly reduced in the LV-mitoAβ group, indicating the inhibition of AHN (Fig. 1g). To further trace the fate of GFP/mitoAβ^+^ cells, cell-type-specific marker proteins and GFP were colabeled. In the control group, most cells were negative for SOX2 and glial fibrillary acidic protein (GFAP), the marker for neural stem cells in the SGZ. In contrast, GFP/mitoAβ^+^ cells were positive for both SOX2 and GFAP but not for the mature differentiated neuron-specific protein calbindin (Fig. 1h, i). Thus, our findings support the hypothesis that neural progenitors with mitochondrial dysfunction fail to properly differentiate into mature neurons, resulting in incomplete inhibition of AHN in vivo.

Although stereotactic surgery activated glial cells in the ipsilateral hemisphere compared to the contralateral hemisphere, mitoAβ led to no significant effect on glial activation, as confirmed by staining for GFAP (astrocytes) or Iba-1 (microglia) (Supplementary Fig. 2a–d). When examining the changes in mitochondrial proteins induced by mitoAβ expression at the tissue level, there were no significant alterations in mitochondrial component proteins, including ATP5A, HSP60, and TOMM20 (Supplementary Fig. 2e). Similarly, staining for TOMM20 in the LV-GFP and LV-mitoAβ groups showed no change in TOMM20 induced by mitoAβ (Supplementary Fig. 2f). Although there were no quantitative changes in the tested mitochondrial proteins, the mitochondrial structural disruption observed in vitro was also examined at the tissue level. We observed mitochondria with a disrupted cristae structure in the hippocampus, where mitoAβ was expressed (Supplementary Fig. 2g).

### AHN deficits mediated by mitoAβ cause memory impairment

Hippocampus-dependent memory was examined to investigate whether inhibition of AHN by mitoAβ is sufficient to cause memory impairment. After 8 weeks of lentiviral transduction, 4-month-old mice were subjected to three different behavioral tests: the passive avoidance test (PAT), Y-maze test, and contextual fear conditioning (CFC) test. The Y-maze test to evaluate the effect of mitoAβ on spatial memory revealed that LV-mitoAβ mice exhibited fewer spontaneous alternations than LV-GFP mice (Fig. 1j). In the CFC test, we observed a significant decrease in the freezing time of LV-mitoAβ mice compared with LV-GFP mice (Fig. 1k). Finally, the PAT to evaluate memory retention showed that LV-GFP and LV-mitoAβ mice exhibited similar learning abilities during the acquisition phase. However, on the following day, LV-mitoAβ mice showed a shorter latency time in the retention trial than LV-GFP mice (Fig. 1l). Taken together, these data suggest that expression of mitoAβ in adult neural stem cells is sufficient to cause AHN deficits and impair hippocampus-dependent cognitive functions.

### AD model mice exhibit AHN deficits in the differentiation stage but not in the proliferation stage

To investigate the alteration of AHN in the hippocampus in AD model mice, we examined the expression of cell markers at the distinct stages of AHN in the SGZ of 5XFAD mice. Neural progenitors and neuroblasts express PCNA at the early and middle stages of AHN; after exiting the proliferation stage, immature neuroblasts differentiate into fully mature neurons expressing DCX in the later stage of AHN^27^. The expression of DCX was found to be significantly reduced in the hippocampus of 6-week-old 5XFAD mice compared to that of wild-type mice, but there was no significant difference in the number of PCNA^+^ cells in the SGZ (Supplementary Fig. 3a–c). Consistent with the PCNA results, the SOX2^+^ cell number in the SGZ (a marker for proliferating neural stem and progenitors) did not differ between 5XFAD and wild-type mice (Supplementary Fig. 3d, e).

To further assess the maturity stages during AHN, we analyzed the morphological features of axons projecting from mature granule cells. We labeled axon fibers with an antibody against synaptoporin, which is exclusively enriched in granule cells in the hippocampus; these cells were specifically labeled along with mossy fibers running in both directions toward the CA3 cell layer (superficial mossy fiber, SMF; and infrapyramidal mossy fiber, IMF)^28^. The synaptoporin intensity, IMF length, and IMF/SMF ratio were decreased in 6-week-old 5XFAD mice compared to wild-type mice, indicating that the maturation of granule cells declined in early AD (Supplementary Fig. 3f, g).

Although extracellular amyloid plaques are observed in the 5XFAD mouse hippocampus beginning at ~16 weeks of age^29^, we detected a nonaggregated form of Αβ and intraneuronal accumulation of Αβ in granule cells in the SGZ in 6-week-old 5XFAD mice, as assessed by Western blot analysis and immunohistochemistry (Supplementary Fig. 3h, i). In rodents, AHN continues actively until ~24 weeks of age; thereafter, the hippocampal niche for AHN typically decreases with age. In humans, continuous AHN occurs throughout the lifespan, resulting in the replacement of most retained granule cells. AHN was still decreased in 20-week-old 5XFAD mice compared to wild-type mice (Supplementary Fig. 4). Taken together, these results indicate that Aβ, not amyloid plaques, impedes AHN by inhibiting the neuronal differentiation process rather than by affecting neural stem/progenitor cells.

### The expression of mitoAβ changed the AHN-associated hippocampal proteome

**Fig. 2:**
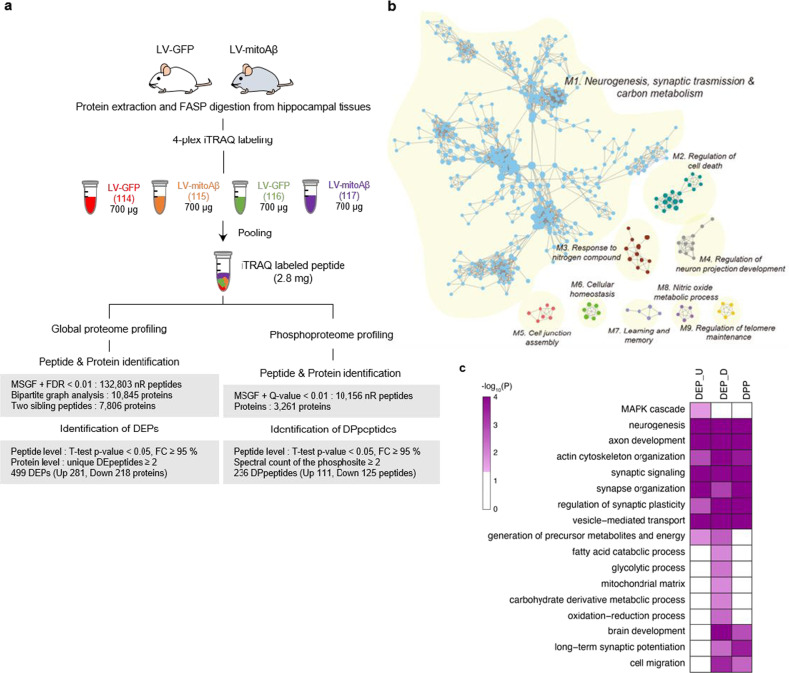
Proteomic analysis reveals that neurogenesis-related pathways exhibited the greatest change in the mitoAβ-expressing hippocampus. **a** Experimental scheme and procedures for global proteome and phosphoproteome analyses of hippocampi from two independent LV-GFP-injected (*n* = 2) and LV-mitoAβ injected mice (*n* = 2). A schematic of the procedure for 4-plex iTRAQ labeling followed by mRP fractionation for global proteome and phosphoproteome profiling is shown. Nonredundant peptides and protein groups identified from the MSGF+ search (FDR <0.01) using global proteome and phosphoproteome data (top), as well as DEPs and DPpeptides identified by statistical testing methods (adjusted *P* < 0.05; bottom), are summarized in the boxes. **b** Functional modules (M1-9) of GOBPs enriched with proteins affected by mitoAβ (up- and downregulated proteins and DPPs). In each module, the node size denotes the number of proteins affected by mitoAβ in the corresponding GOBP, and the edges indicate that the corresponding pair of GOBPs has a significant similarity score. **c** GO terms associated with the phenotypes altered by mitoAβ in the “neurogenesis, synaptic transmission, and carbon metabolism” module. The colors in the heatmap denote the significance (*P* value) of GOBPs enriched with upregulated (DEP_U) and downregulated (DEP_D) proteins and DPPs. The color bar represents the gradient of −log_10_(*P*), where *P* is the enrichment *P* value determined with DAVID software.

To identify biological pathways altered by mitoAβ, we performed global proteomic and phosphoproteomic profiling of hippocampal tissues from LV-GFP- and LV-mitoAβ-injected mice using liquid chromatography (LC)-tandem mass spectrometry (LC‒MS/MS) analysis (Fig. 2a). We identified 499 differentially expressed proteins (DEPs; 281 upregulated and 218 downregulated, *P* < 0.05) and 236 differentially phosphorylated proteins (DPPs; 111 upregulated and 125 downregulated, *P* < 0.05) in LV-mitoAβ hippocampal tissues compared to LV-GFP controls (Supplementary Data 1).

We next identified 586 Gene Ontology biological processes (GOBP) enriched with the three sets of proteins: (1) the 281 upregulated proteins, (2) the 218 downregulated proteins, and (3) the 191 DPPs (Supplementary Data 1). To investigate the functional modules affected by mitoAβ, we built GOBP association networks based on relationships among the 586 GOBPs as described in the Materials and Methods. The GOBP networks revealed the following functional modules affected by mitoAβ (Fig. 2b): (1) neurogenesis, synaptic transmission, and carbon metabolism; (2) regulation of cell death; (3) response to nitrogen compound; (4) regulation of neuron projection development; (5) cell junction; (6) cellular homeostasis; (7) learning and memory; (8) nitric oxide metabolic process; and (9) regulation of telomere maintenance. Interestingly, among these nine functional modules, the “neurogenesis, synaptic transmission, and carbon metabolism” module was found to be most significantly affected by mitoAβ. Among the GOBPs in this module, we then selected GOBPs that were deemed to be consistent with the phenotypes observed in the mitoAβ-expressing hippocampus and adult neural stem cells. Among these selected GOBPs, neurogenesis, axon development, and cytoskeleton organization were commonly enriched with all three sets of proteins, whereas brain development, long-term synaptic potentiation, and cell migration were specifically enriched with the downregulated proteins (Fig. 2c). Furthermore, we observed specific enrichment of the term mitochondrion with the downregulated proteins (Fig. 2c). These results support our finding that mitoAβ strongly dysregulates AHN at the global proteome level.

### Mitochondrial dysfunction has no effect on the characteristics of neural progenitors

**Fig. 3:**
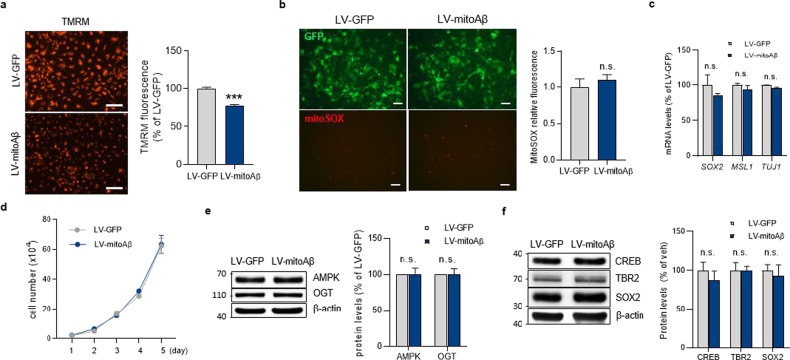
Mitochondrial dysfunction does not affect the characteristics of neural progenitors. **a** Representative images and quantification of the mitochondrial membrane potential, as determined by staining with TMRM, of the ReNcell CX human neural progenitor cell line transduced with LV-GFP or LV-mitoAβ. Scale bar, 100 μm. Unpaired *t* test, *n* = 3 in each group. **b** Live-cell images of mitochondria-derived ROS in LV-GFP and LV-mitoAβ neural progenitors, as detected by MitoSOX staining. Scale bar, 100 μm. Unpaired *t* test, *n* = 5 in each group. **c** The levels of neural progenitor characteristic genes in LV-GFP and LV-mitoAβ neural progenitors, as measured by RT‒PCR. Unpaired *t* test, *n* = 3 in each group. **d** Proliferation ability of LV-GFP and LV-mitoAβ neural progenitors, as assessed by the cell number on each day. Unpaired *t* test for each day, *n* = 3 in each group. **e** The expression levels of the epigenetic regulators AMPK and OGT in LV-mitoAβ neural progenitors. Data for each protein were normalized to the level of β-actin and the mean value in the LV-GFP group. Unpaired *t* test, *n* = 7 in each group. **f** The expression levels of the transcription factors CREB, TRB2, and SOX2, which are required for neural differentiation, in LV-GFP and LV-mitoAβ neural progenitors. Unpaired *t* test, *n* = 3 in each group. All results are presented as the means ± SEMs. ***P < 0.001.

We investigated the characteristics of neural progenitors expressing mitoAβ. MitoAβ lowered the mitochondrial membrane potential (Fig. 3a) but did not alter the mitochondria-derived ROS level (Fig. 3b). MitoAβ expression did not change the levels of characteristic genes of neural progenitors (*SOX2*, *MSL1*, *TUJ1*) (Fig. 3c) or the proliferation properties of neural progenitors (Fig. 3d). Epigenetic regulation of gene expression can be controlled by the metabolic status of a cell and the resulting metabolites^30^. AMP-activated protein kinase (AMPK) is responsive to the ATP/AMP ratio and phosphorylates histones, while O-linked N-acetylglucosamine transferase (OGT) is associated with the glucose and hexosamine biosynthetic pathways, and it glycosylates histones^30–32^. We examined the expression of epigenetic modulators associated with cellular metabolism (AMPK and OGT). MitoAβ had no effect on the protein levels of AMPK and OGT (Fig. 3e). In addition, there were no changes in the transcription factors required for neuronal differentiation due to mitochondrial dysfunction (Fig. 3f). Thus, resistance to mitochondrial dysfunction depends on the stage of AHN and has a decisive effect on differentiated cells rather than neural progenitors.

### Mitochondrial dysfunction in neural progenitors induces KDM5A degradation

Next, we sought to identify upstream regulators that mediate the inhibition of AHN due to mitochondrial damage in neural progenitors. Using a ChIP-sequencing database (chip-atlas.org), we identified potential transcription factors and epigenetic regulators for a total of 499 DEPs (281 upregulated DEPs and 218 downregulated DEPs; Fig. 2a) affected by mitoAβ expression in the SGZ of the hippocampus. Based on ChIP-seq binding enrichment analysis, we identified 49 potential regulators involved in hippocampal proteomic changes (Supplementary Fig. 5a). Since it was confirmed that hippocampal neurogenesis is blocked at the neural progenitor stage by mitoAβ in vitro and in vivo and cannot proceed to the next stage, we narrowed down the candidates by considering the expression pattern of 49 potential regulators based on RNA-seq datasets for in vitro neuronal differentiation over a time course^33^. Hierarchical clustering analysis grouped the 49 regulators into five distinct categories: (1) ES cell/early differentiation, (2) early differentiation, (3) middle differentiation, (4) late differentiation, and (5) ES cell (Supplementary Fig. 5b and Supplementary Data 2). The regulators in category 2 exhibited high expression levels in neural progenitors at the early stage of neuronal differentiation. The last step for selecting a target protein was to screen the candidates related to “mitochondria” and “metabolism” among the seven regulators in category 2. Interestingly, only lysine demethylase 5A (KDM5A) is an epigenetic regulator that has been reported to be associated with metabolism and mitochondrial biogenesis^34,35^. Moreover, KDM5A has been reported to be involved in the differentiation of different cell types or tissues^34,36,37^. Considering the relationship among KDM5A, mitochondria, and cell differentiation, we hypothesized that KDM5A expression could be adversely affected by mitochondrial dysfunction in neural progenitors, resulting in the inhibition of neuronal differentiation during hippocampal neurogenesis.

**Fig. 4:**
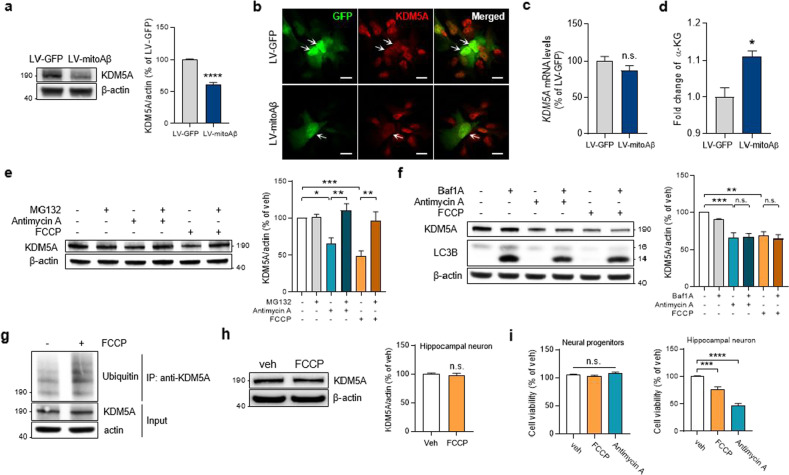
Mitochondrial dysfunction induces KDM5A degradation in neural progenitors. **a** KDM5A protein levels in ReNcell CX neural progenitors transduced with LV-GFP or LV-mitoAβ. Unpaired *t* test, *n* = 3 in each group. **b** Immunostaining for KDM5A in GFP^+^ and GFP/mitoAβ^+^ neural progenitors. The arrows indicate lentivirally transduced cells. Scale bar, 20 μm. **c** Quantitative analysis of *KDM5A* mRNA levels in LV-GFP and LV-mitoAβ neural progenitors. Unpaired *t* test, *n* = 3 in each group. **d** α-KG levels measured in LV-GFP and LV-mitoAβ neural progenitors. Unpaired *t* test, *n* = 3 in each group. **e**, **f** KDM5A protein levels in neural progenitors cotreated with a mitochondrial dysfunction modulator (antimycin A or FCCP; 3 μM, 12 h for each drug) and either MG132 (5 μM, 12 h), a proteasome inhibitor, or bafilomycin A (10 nM, 12 h), an autophagy inhibitor. One-way ANOVA, *n* = 4–5 in each group. **g** Ubiquitination of KDM5A in neural progenitors treated with FCCP (3 μM, 12 h). **h** Western blot analysis and quantification of KDM5A protein levels in hippocampal neurons (DIV21) treated with FCCP (3 μM, 12 h). Unpaired *t* test, *n* = 10 in each group. **i** The effect of mitochondrial dysfunction modulators (antimycin A and FCCP; 3 μM, 12 h for each drug) on the viability of two different cell types, neural progenitors and hippocampal neurons (DIV21), as assessed by an MTT assay. One-way ANOVA, *n* = 6–8 in each group. All results are presented as the means ± SEMs. *P < 0.05; **P < 0.01; ***P < 0.001; ****P < 0.0001.

To further elucidate the molecular mechanisms underlying the impairment of neuronal differentiation by mitochondrial dysfunction, we examined KDM5A expression in neural progenitors expressing mitoAβ. In contrast to other transcription factors (Fig. 3e, f), mitoAβ expression decreased KDM5A protein levels (Fig. 4a, b), but it led to no decrease in *KDM5A* mRNA levels (Fig. 4c). Jumonji-C domain-containing histone demethylases (JHDMs), such as KDM5 family members, utilize alpha-ketoglutarate (α-KG) as a cofactor and promote the demethylation of histones^38^. Since the α-KG level was increased by mitoAβ expression in neural progenitors, we confirmed that the decreased KDM5A protein level was not due to insufficiency of a cofactor (Fig. 4d). This is consistent with previous reports that the activity of the α-KG dehydrogenase complex is inhibited by Aβ in AD brains, thus increasing the α-KG level^39,40^. Next, we examined KDM5A levels in neural progenitors following treatment with mitochondrial dysfunction modulators that lower the mitochondrial membrane potential, similar to the effect of mitoAβ (Supplementary Fig. 6). Consistent with the effect of mitoAβ, KDM5A was degraded by treatment with mitochondrial dysfunction modulators (antimycin A and FCCP), particularly via the ubiquitin‒proteasome degradation system, and this degradation was rescued by the proteasome inhibitor MG132 (Fig. 4e). However, the decrease in KDM5A was not rescued by the autophagy flux inhibitor bafilomycin A (Fig. 4f). In addition, supported by the increased ubiquitination of KDM5A after FCCP treatment (Fig. 4g), our results indicate that mitochondrial dysfunction activates the ubiquitin‒proteasome system and leads to KDM5A degradation in neural progenitors.

Interestingly, FCCP treatment of mouse hippocampal neurons did not induce the degradation of KDM5A (Fig. 4h). Moreover, both FCCP and antimycin A failed to significantly affect the viability of neural progenitors but caused cytotoxicity in mouse hippocampal neurons (Fig. 4i). These results suggest that whether resistance to mitochondrial damage or the mitochondria-based signaling pathway regulates the degradation of KDM5A may differ depending on the cell type.

### Degradation of KDM5A is induced by various reactions from mitochondrial damage

The fundamental function of mitochondria is to regulate calcium homeostasis in the cytoplasm^41^. When mitochondrial damage interferes with the buffering of calcium ions, cytosolic calcium levels increase and activate various calcium ion-regulated kinases, including Ca^2+^/calmodulin-dependent protein kinase II (CaMKII). Neural progenitors treated with an inhibitor of ATP synthase, oligomycin A, exhibited activation of CaMKII and downregulation of KDM5A. When these cells were treated with both oligomycin A and an inhibitor of CaMKII (KN-93), KDM5A protein levels were restored, indicating that the signaling pathway through which oligomycin A decreases KDM5A depends on calcium and CaMKII (Supplementary Fig. 7a, b). Similar to NPCs treated with mitoAβ expression and FCCP treatment, NPCs treated with oligomycin A expressed less BDNF and MEF2A during differentiation (Supplementary Fig. 7c). However, as there were no changes in the levels of mitochondrial protein and transcription factors required for differentiation after oligomycin A treatment, it was confirmed that the KDM5A-specific decrease was caused by mitochondrial damage (Supplementary Fig. 7d, e). FCCP also promoted downregulation of KDM5A, but KN-93 did not reverse the decrease in the KDM5A protein level induced by FCCP (Supplementary Fig. 7f). These results indicate that mitochondrial dysfunction appears to induce KDM5A degradation through various signaling pathways.

### KDM5A in neural progenitors participates in neuronal differentiation

**Fig. 5:**
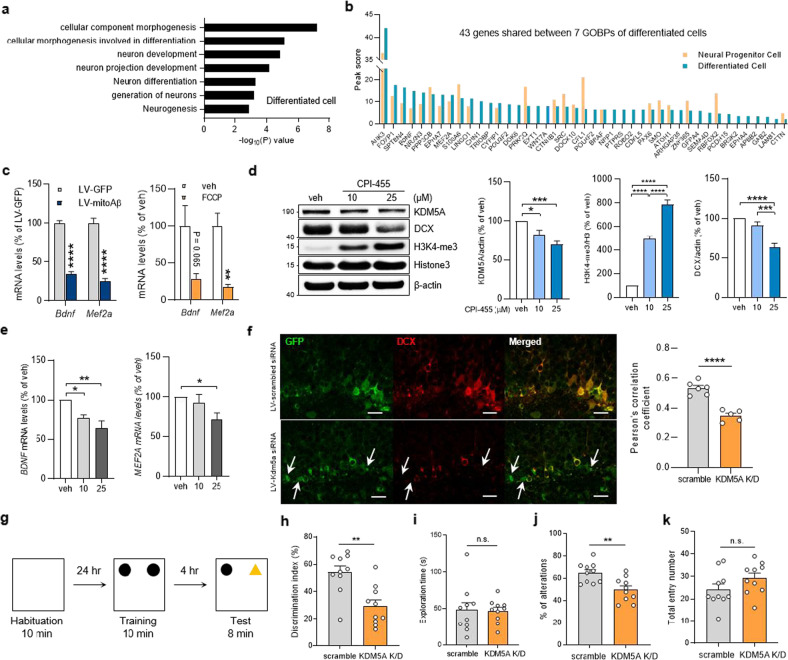
KDM5A regulates neuronal differentiation in neural progenitors. **a** Top seven GOBPs enriched with KDM5A target genes in differentiated cells (5 days). The significance of enrichment is shown as the −log_10_(*P*) value, where *P* is the enrichment *P* value. **b** Peak scores of 43 genes shared between the seven GOBPs of KDM5A target genes in neural progenitors and differentiated cells. **c** Quantitative analysis of the mRNA levels of the target genes *BDNF* and *MEF2A* in LV-GFP and LV-mitoAβ differentiated cells (12 days). Unpaired *t* test, *n* = 3–6 in each group. **d** Western blot analysis of KDM5A, DCX, and Histone3 in differentiated cells (12 days) treated with CPI-455, a KDM5A inhibitor, to promote differentiation. Data for each protein were normalized to the level of β-actin and the mean value in the vehicle group. One-way ANOVA, *n* = 8 in each group. **e** Quantitative analysis of *BDNF* and *MEF2A* mRNA levels in differentiated cells (12 days) treated with CPI-455 to promote differentiation. One-way ANOVA, *n* = 6 in each group. **f** Immunostaining for DCX and GFP (transduced cells) in the SGZ of mice injected with LV-scramble siRNA and LV-*Kdm5a* siRNA. Quantitative analysis of Pearson correlation coefficients between the GFP and DCX signals in the LV-scramble siRNA and LV-*Kdm5a*-siRNA groups. Unpaired *t* test, *n* = 6 (scramble) or 5 (*Kdm5a* siRNA) mice. Scale bar, 20 μm. **g** Experimental procedure for the NOR test. **h**, **i** Discrimination index and total exploration time in the NOR test. Unpaired *t* test, *n* = 10 in each group. **j**, **k** The percentage of alternations and total entry number in the Y-maze test. Unpaired *t* test, *n* = 10 in each group. All results are presented as the means ± SEMs. *P < 0.05; **P < 0.01; ***P < 0.001; ****P < 0.0001.

To further explore the role of KDM5A in neuronal differentiation, its target genes in differentiated cells were investigated by chromatin immunoprecipitation sequencing (ChIP-seq) (Supplementary Data 3). Notably, according to GOBP enrichment analysis, the target genes of KDM5A were involved in neuronal differentiation-associated processes, such as cellular component morphogenesis, cellular morphogenesis involved in differentiation, and neuron differentiation (Fig. 5a and Supplementary Data 4). Forty-three genes, including *BDNF* and *MEF2A*, belonging to 7 common GOBPs (Fig. 5a) were transcribed more actively in differentiated cells than in neural progenitors (Fig. 5b). The mRNA levels of both *BDNF* and *MEF2A* mRNA, previously known to be important for neurogenesis, were reduced both in mitoAβ-expressing and FCCP-treated differentiated cells (Fig. 5c).

We next used the KDM5A inhibitor CPI-455 to specifically examine whether a decrease in KDM5A activity impairs neuronal differentiation. We found that KDM5A inhibition in neural progenitors through CPI-455 treatment reduced DCX expression in differentiated cells by increasing H3K4 methylation levels (Fig. 5d). The levels of the KDM5A target genes *BDNF* and *MEF2A* were also reduced by CPI-455 treatment as well as by mitoAβ expression and mitochondrial stress-inducing drugs (Fig. 5e).

**Fig. 6:**
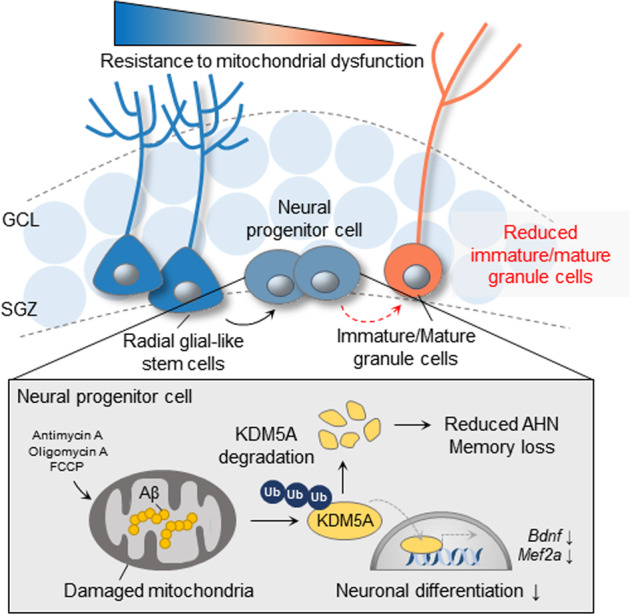
Graphical summary. Graphical summary showing that Aβ-induced mitochondrial dysfunction in neural progenitors causes KDM5A degradation, thereby inhibiting neuronal differentiation and compromising cognitive function.

To verify the effect of *KDM5A* knockdown on neuronal differentiation, we infused *Kdm5a*-siRNA lentiviruses into the DGs of 8-week-old C57BL/6 mice and traced the final fate of *Kdm5a*-knockdown cells during AHN. Four weeks after viral transduction, compared to the scramble siRNA-transduced group, most of the *Kdm5a*-knockdown cells in the SGZ did not exhibit DCX costaining (Fig. 5f). We next used three different behavioral tests to examine the effect of *Kdm5a* knockdown on cognitive functions. In the novel object recognition (NOR) test, the *Kdm5a*-knockdown group showed a lower discrimination index than the control group (Fig. 5g, h). There was no significant difference in the total exploration time between the two groups (Fig. 5i). In addition, mice in the *Kdm5a*-knockdown group showed significantly impaired spatial memory in the Y-maze test (Fig. 5j, k). These results suggest that knockdown of KDM5A impairs neuronal differentiation in vivo, as it does in vitro, and is sufficient to cause cognitive impairment. Taken together, our data suggest that mitochondrial dysfunction-induced KDM5A degradation in neural progenitors dysregulates the transcription of genes essential for neuronal differentiation, such as *BDNF* and *MEF2A*, thereby inhibiting the differentiation of these cells into neurons (Fig. 6).

## Discussion

AHN occurs throughout the human lifespan and is reported to be drastically lower in AD patients and AD model mice than in their non-AD counterparts, but relatively little is known about the molecular mechanisms underlying the loss of AHN in AD^42^. In this study, we define that the loss of AHN in AD is associated with amyloid pathology and mitochondrial dysfunction caused by Aβ. In AD patient brains, AD pathology has different effects on the various stages of AHN and dysregulates neuronal differentiation rather than affecting neural stem/progenitors^5^. As observed in AD patients, we found that DCX^+^ cells were markedly reduced in the hippocampus in 6-week-old 5XFAD mice even before the initiation of amyloid plaque deposition, whereas there were no significant differences in PCNA^+^ and SOX2^+^ cells, indicating that neuronal differentiation during AHN is vulnerable to soluble Aβ beginning in the early stage of AD. Aβ is excessively produced beginning at an early age in 5XFAD model mice, particularly in the hippocampus, subiculum, and frontal cortex^29^. This model is characterized by early detection of intraneuronal Aβ aggregates starting at 6 weeks of age. These early pathological features predispose the SGZ region, where adult neural stem/progenitor cells are localized, and adult hippocampal neurogenesis occurs, to deficits in hippocampal neurogenesis^43^.

Mitochondrial regulation in stem cells is crucial for AHN and decisions related to stem cell fate^10,44^. Considering the hippocampal niche where AHN occurs, we hypothesized that in AD brains, mitochondrial dysfunction in neural stem/progenitors mediated by Aβ dysregulates neuronal differentiation, thereby leading to AHN deficits. First, we found that neuronal differentiation of neural progenitors was suppressed by pharmacological inhibition of mitochondrial functions. Next, using previously developed mitoAβ^26^, which accumulates within mitochondria and induces mitochondrial dysfunction, we showed that the expression of mitoAβ in neural progenitors blocked the normal completion of the neuronal differentiation process in vitro and in vivo. Our results strongly support the idea that mitochondria in neural progenitors are central regulators of neuronal differentiation. Regarding the quality control of damaged or superfluous mitochondria, mitophagy plays a distinct role in maintaining the stemness of proliferating cells^45,46^. In addition, balanced mitochondrial fission and accompanying mitophagy are critical for regulating cell death in hippocampal neural progenitors^47^. Mitophagy is compromised in AD and implicated in memory loss in AD^48–50^. Although there are a few studies on mitophagy changes in neural progenitors in AD, failed mitochondrial quality control in the SGZ by amyloid pathology may impact and accelerate the inhibition of neuronal differentiation of neural progenitors.

When neural progenitors in the SGZ were subjected to mitoAβ expression in vivo, changes in the hippocampal proteome were accompanied by a decrease in AHN. We predicted the potential upstream regulators involved, including transcription factors and epigenetic regulators, among the DEPs after mitoAβ expression. Considering the RNA expression levels of regulators during AHN and their association with mitochondria, KDM5A was finally selected as a mediator to indicate mitochondrial dysfunction in neural progenitors and regulate neuronal differentiation. KDM5A degradation via the ubiquitin‒proteasome system was induced by mitoAβ expression as well as treatment with mitochondrial stress-inducing drugs. It is worth noting that mitochondrial dysfunction may contribute to regulating the degradation of epigenetic regulators involved in AHN. Both Aβ accumulation in mitochondria and indirect mitochondrial damage caused by signaling through various pathways in the AD environment can lead to degradation of KDM5A, resulting in AHN deficits. Furthermore, we revealed that KDM5A is involved in the transcriptional regulation of genes important for neurogenesis and morphogenesis in differentiating cells. During the differentiation of neural progenitors, KDM5A promotes the transcription of *BDNF* and *MEF2A*, which are required for AHN^51,52^. Pharmacological and genetic reduction of KDM5A expression in neural progenitors decreased *BDNF* and *MEF2A* transcription during differentiation, inhibited neural differentiation, and caused memory deficits.

We herein report that mitochondrial damage in neural progenitors inhibits their differentiation into mature neurons. Moreover, mitochondrial dysfunction promotes the degradation of the epigenetic regulator KDM5A, which regulates neuronal differentiation, leading to AHN deficits. Our collective results imply that restoration of mitochondrial function in the hippocampal niche of AD brains may protect against the decline in AHN caused by AD pathogenesis, and this finding may suggest new approaches for the treatment of AD.

## Supplementary information

Supplementary information

Dataset 1

Dataset 2

Dataset 3

Dataset 4

## References

[1] Kempermann G, Song H, Gage FH (2015). Neurogenesis in the adult hippocampus.. Cold Spring Harb. Perspect. Biol..

[2] Ming GL, Song H (2011). Adult neurogenesis in the mammalian brain: significant answers and significant questions. Neuron.

[3] Gage FH (2019). Adult neurogenesis in mammals. Science.

[4] von Bohlen Und Halbach O (2007). Immunohistological markers for staging neurogenesis in adult hippocampus. Cell Tissue Res..

[5] Moreno-Jimenez EP (2019). Adult hippocampal neurogenesis is abundant in neurologically healthy subjects and drops sharply in patients with Alzheimer's disease. Nat. Med..

[6] Choi SH (2018). Combined adult neurogenesis and BDNF mimic exercise effects on cognition in an Alzheimer's mouse model. Science.

[7] Demars M, Hu YS, Gadadhar A, Lazarov O (2010). Impaired neurogenesis is an early event in the etiology of familial Alzheimer's disease in transgenic mice. J. Neurosci. Res..

[8] Rodriguez JJ (2008). Impaired adult neurogenesis in the dentate gyrus of a triple transgenic mouse model of Alzheimer's disease. PLoS ONE.

[9] Folmes CD (2011). Somatic oxidative bioenergetics transitions into pluripotency-dependent glycolysis to facilitate nuclear reprogramming. Cell Metab..

[10] Khacho M, Harris R, Slack RS (2019). Mitochondria as central regulators of neural stem cell fate and cognitive function. Nat. Rev. Neurosci..

[11] Iwata R, Casimir P, Vanderhaeghen P (2020). Mitochondrial dynamics in postmitotic cells regulate neurogenesis. Science.

[12] Khacho M (2016). Mitochondrial dynamics impacts stem cell identity and fate decisions by regulating a nuclear transcriptional program. Cell Stem Cell.

[13] Zhang H (2016). NAD(+) repletion improves mitochondrial and stem cell function and enhances life span in mice. Science.

[14] Beckervordersandforth R (2017). Role of mitochondrial metabolism in the control of early lineage progression and aging phenotypes in adult hippocampal neurogenesis. Neuron.

[15] Liu Z, Butow RA (2006). Mitochondrial retrograde signaling. Annu. Rev. Genet..

[16] Kim KH, Son JM, Benayoun BA, Lee C (2018). The mitochondrial-encoded peptide MOTS-c translocates to the nucleus to regulate nuclear gene expression in response to metabolic stress. Cell Metab..

[17] Cha MY, Kim DK, Mook-Jung I (2015). The role of mitochondrial DNA mutation on neurodegenerative diseases. Exp. Mol. Med..

[18] Sidler C, Kovalchuk O, Kovalchuk I (2017). Epigenetic regulation of cellular senescence and aging. Front. Genet..

[19] Zou C, Mallampalli RK (2014). Regulation of histone modifying enzymes by the ubiquitin-proteasome system. Biochim. Biophys. Acta.

[20] Yu SW (2008). Autophagic death of adult hippocampal neural stem cells following insulin withdrawal. Stem Cells.

[21] Ha S (2019). Autophagy mediates astrogenesis in adult hippocampal neural stem cells. Exp. Neurobiol..

[22] Oki S (2018). ChIP-Atlas: a data-mining suite powered by full integration of public ChIP-seq data. EMBO Rep..

[23] Demine S, Renard P, Arnould T (2019). Mitochondrial uncoupling: a key controller of biological processes in physiology and diseases. Cells.

[24] Penefsky HS (1985). Mechanism of inhibition of mitochondrial adenosine triphosphatase by dicyclohexylcarbodiimide and oligomycin: relationship to ATP synthesis. Proc. Natl Acad. Sci. USA.

[25] Pagani L, Eckert A (2011). Amyloid-Beta interaction with mitochondria. Int. J. Alzheimers Dis..

[26] Cha MY (2012). Mitochondria-specific accumulation of amyloid beta induces mitochondrial dysfunction leading to apoptotic cell death. PLoS ONE.

[27] Herrup K, Yang Y (2007). Cell cycle regulation in the postmitotic neuron: oxymoron or new biology?. Nat. Rev. Neurosci..

[28] Singec I (2002). Synaptic vesicle protein synaptoporin is differently expressed by subpopulations of mouse hippocampal neurons. J. Comp. Neurol..

[29] Oakley H (2006). Intraneuronal beta-amyloid aggregates, neurodegeneration, and neuron loss in transgenic mice with five familial Alzheimer's disease mutations: potential factors in amyloid plaque formation. J. Neurosci..

[30] Lu C, Thompson CB (2012). Metabolic regulation of epigenetics. Cell Metab..

[31] Dehennaut V, Leprince D, Lefebvre T (2014). O-GlcNAcylation, an epigenetic mark. focus on the histone code, TET family proteins, and polycomb group proteins. Front. Endocrinol..

[32] Bungard D (2010). Signaling kinase AMPK activates stress-promoted transcription via histone H2B phosphorylation. Science.

[33] Pataskar A (2016). NeuroD1 reprograms chromatin and transcription factor landscapes to induce the neuronal program. EMBO J..

[34] Varaljai R (2015). Increased mitochondrial function downstream from KDM5A histone demethylase rescues differentiation in pRB-deficient cells. Genes Dev..

[35] Liu X, Secombe J (2015). The histone demethylase KDM5 activates gene expression by recognizing chromatin context through Its PHD reader motif. Cell Rep..

[36] Lopez-Bigas N (2008). Genome-wide analysis of the H3K4 histone demethylase RBP2 reveals a transcriptional program controlling differentiation. Mol. Cell.

[37] Peng JC (2009). Jarid2/Jumonji coordinates control of PRC2 enzymatic activity and target gene occupancy in pluripotent cells. Cell.

[38] Tsukada Y (2006). Histone demethylation by a family of JmjC domain-containing proteins. Nature.

[39] Casley CS, Canevari L, Land JM, Clark JB, Sharpe MA (2002). Beta-amyloid inhibits integrated mitochondrial respiration and key enzyme activities. J. Neurochem..

[40] Gibson GE (1998). Alpha-ketoglutarate dehydrogenase in Alzheimer brains bearing the APP670/671 mutation. Ann. Neurol..

[41] Giorgi C, Marchi S, Pinton P (2018). The machineries, regulation and cellular functions of mitochondrial calcium. Nat. Rev. Mol. Cell Biol..

[42] Babcock KR, Page JS, Fallon JR, Webb AE (2021). Adult hippocampal neurogenesis in aging and Alzheimer's disease. Stem Cell Rep..

[43] Reilly JF (2003). Amyloid deposition in the hippocampus and entorhinal cortex: quantitative analysis of a transgenic mouse model. Proc. Natl Acad. Sci. USA.

[44] Wanet A, Arnould T, Najimi M, Renard P (2015). Connecting mitochondria, metabolism, and stem cell fate. Stem Cells Dev..

[45] Ho TT (2017). Autophagy maintains the metabolism and function of young and old stem cells. Nature.

[46] Leeman DS (2018). Lysosome activation clears aggregates and enhances quiescent neural stem cell activation during aging. Science.

[47] Woo HN (2021). miR-351-5p/Miro2 axis contributes to hippocampal neural progenitor cell death via unbalanced mitochondrial fission. Mol. Ther. Nucleic Acids.

[48] Fang EF (2019). Mitophagy inhibits amyloid-beta and tau pathology and reverses cognitive deficits in models of Alzheimer's disease. Nat. Neurosci..

[49] Kobro-Flatmoen A (2021). Re-emphasizing early Alzheimer's disease pathology starting in select entorhinal neurons, with a special focus on mitophagy. Ageing Res. Rev..

[50] Xie C (2022). Amelioration of Alzheimer's disease pathology by mitophagy inducers identified via machine learning and a cross-species workflow. Nat. Biomed. Eng..

[51] Bath KG, Akins MR, Lee FS (2012). BDNF control of adult SVZ neurogenesis. Dev. Psychobiol..

[52] Li H (2008). Transcription factor MEF2C influences neural stem/progenitor cell differentiation and maturation in vivo. Proc. Natl Acad. Sci. USA.

