# Supplementary material for: Aβ-induced mitochondrial dysfunction in neural progenitors controls KDM5A to influence neuronal differentiation
Source: Exp Mol Med. 2022 Sep 2;54(9):1461–71. doi: 10.1038/s12276-022-00841-w (PMC9534996; doi:10.1038/s12276-022-00841-w)
Supplement: Supplementary file 1 — Supplementary information [file 12276_2022_841_MOESM1_ESM.pdf]

Supplementary Information

Aβ-induced mitochondrial dysfunction in neural progenitors controls KDM5A to influence neuronal differentiation

Dong Kyu Kim[1,2], Hyobin Jeong[3,4], Jingi Bae[5], Moon-Yong Cha[6], Moonkyung Kang[7], Dongjin Shin[1], Shinwon Ha[8], Seung Jae Hyeon[9], Hokeun Kim[5], Kyujin Suh[1,2], Mi-Sun Choi[10], Hoon Ryu[9], Seong-Woon Yu[8], Jong-Il Kim[1,11], Yeon-Soo Kim[7], Sang-Won Lee[5*], Daehee Hwang[4*], Inhee Mook-Jung[1,2*]

Contents for Supplementary information

1. Supplementary Materials and Methods
2. Supplementary Table 1
3. Supplementary Figures 1-7
4. References

Supplementary Materials and Methods

Construction of pLentiSyn1.41g-mitoAβ

MitoAβ sequence was designed and constructed as previously described[20]. To prepare the pLentiSyn1.41g-mitoAβ construct, a 222-bp of mitoAβ gene was amplified from pcDNA-mitoAβ by PCR using the following primers: mitoAβ-*EcoR* V-F, 5‘-CGGATATCATGTCCGTCCTGACGCCGCT-3’ and mitoAβ-*EcoR* V-R, 5‘-GCGATATC CTACGCTATGACAACACCGC-3’. The resulting PCR fragment was digested with *EcoR* V and ligated into a lentiviral vector pLentiSyn1.41g under the control of the murine cytomegalovirus immediate early gene promoter. The pLentiSyn1.41g encodes GFP which is expressed by human synapsin 1 promoter.

Production of pLentiSyn1.41g-mitoAβ

Human embryonic kidney (HEK) 293T cells were cultured in Dulbecco’s modified Eagle’s medium (Hyclone Laboratories Inc) with 10 % fetal bovine serum (Gibco-BRL, Invitrogen) and maintained in a 5 % CO_2 incubator at 37 °C. The lentivirus particles were produced by co-transfection of HEK 293T cells with the following three plasmids using Lipofectamine Plus (Invitrogen): VSV-G, *gag-pol*, and pLentiSyn1.41g-mitoAβ. At 48 hrs post-transfection, culture supernatants containing virus particles were collected and clarified with a 0.45 μm membrane filter (Nalgene) and stored in a -70 °C deep-freezer immediately. Titers were determined with p24 enzyme-linked immunosorbent assays (Perkin-Elmer Life Science) or western blot analyses using a monoclonal anti-p24 antibody (obtained from the AIDS Research and Reference Reagent Program, National Institutes of Health, Bethesda, MD, USA). In our routine preparation, the titers were ≒ 10^7 transduction units (TU) per mL without further concentration. For stereotaxic injection, the lentivirus particles were concentrated by ultracentrifugation on a 20 % sucrose cushion (2 hrs at 50,000 *g*) at 4 °C.

Mitochondria fractionation

In order to separate the cytoplasm and mitochondria, cells were homogenized in the solution with 20 mM HEPES, pH 7.5, 250 mM sucrose, 20 mM KCl, 1.5 mM MgCl2, 1 mM EDTA, 1 mM dithiothreitol, and 1 mM phenylmethylsulfonyl fluoride by using a glass Dounce homogenizer. Cell lysates were centrifuged at 600 g for 10 min at 4 °C to remove undissolved cell debris and nuclei. The supernatant was again centrifuged at 7,000 g for 10 min at 4 °C, and the resulting pellet was collected as a mitochondria-enriched fraction.

α-ketoglutarate assay

The amount of α-ketoglutarate was measured by using the α-ketoglutarate assay kit (Abcam, ab83431) following manufacturer instructions. In brief, ReN CX cells expressing GFP or mitoAβ were homogenized in 500 µl Alpha KG assay buffer by pipetting. Cell lysates were centrifuged at 13,000 g for 5 min, and the resulting supernatant was used for further procedures. Cell lysates were diluted in the perchloric acid solution (PCA; final concentration, 1 M) and centrifuged at 13,000g for 2 min to deplete proteins. Next, 2 M KOH solution (34 % of sample volume) was added to precipitate remained PCA, and the neutralized sample was centrifuged at 13,000 g for 15 min. 50 µl of α-ketoglutarate standard or cell lysates were mixed with the reaction mixture of a probe and converting enzyme. After incubation at 37 °C for 30 min, the results were measured on a PowerWave XS microplate reader (BioTek) at OD570 nm.

Transmission Electron Microscope imaging

Hippocampal tissues and HT22 cells expressing mitoAβ were fixed for one day in a mixture of cold 2.5 % glutaraldehyde diluted in 0.1 M phosphate buffer (pH 7.2) and 2 % paraformaldehyde diluted in 0.1 M phosphate or cacodylate buffer (pH 7.2). After the reaction, fixed tissues and cells were immersed in epoxy resin, and the samples were loaded into capsules and polymerized for 12 hrs at 38 °C and 48 hrs at 60 °C. Samples were cut to a thickness of 65 nm using an ultramicrotome (RMC MT-XL; RMC

Products), stained with 4 % uranyl acetate and 4 % lead citrate. Stained samples were imaged by transmission electron microscopy (JEM-1400; Japan) at 80 Kv.

Chromatin immunoprecipitation (ChIP)

ReN CX cells or differentiated cells (after 5 days) were fixed with 1 % formaldehyde at room temperature for 10 min. After cross-linking reactions, the fixation was stopped by adding 1 M glycine solution to be at a final concentration of 125 mM. The solution was centrifuged at 3,000 rpm for 5 min, and the resulting pellet was then washed 3 times with ice-cold phosphate-buffered saline (PBS). Cell pellets were suspended with lysis buffer (sc-45000, Santa Cruz) and centrifuged at 3,000 rpm for 5 min. Crude nuclear extract was re-suspended with high salt lysis buffer (sc-45001, Santa Cruz) and sonicated 15 times by 30 s burst, each followed by 1 min incubation on ice. The sample was centrifuged at 14,000 rpm for 10 min, and then the supernatants containing chromatin were diluted 1:10 with the dilution buffer containing 0.01 % SDS, 1.1 % Tritonx-100, 1.2 mM EDTA, 16.7 mM Tris pH 8.1, 167 mM NaCl, and proteinase inhibitor cocktail. For preclearing the chromatin samples, 50 µl Protein A/G PLUS-Agarose beads (sc-2003, Santa Cruz) were added to the samples and incubated at 4 °C for 30 min. To precipitate agarose beads, the chromatin solution was centrifuged at 12,000 rpm for 20 s. 5 – 10 µg antibodies were added in the precleared chromatin solution and incubated overnight at 4 °C. On the following day, 50 µl Protein A/G PLUS-Agarose beads were added and incubated at 4 °C for 2 hrs. To collect the beads, the solution was centrifuged at 12,000 rpm for 20 s. After the beads were washed with high salt lysis buffer and wash buffer (sc-45002, Santa Cruz), the beads were suspended with 500 µl of elution buffer with 20 µl of 5 M NaCl. Cross-linking was reversed by heating at 65 °C for 4 hrs. To isolate DNA fragments, the supernatants were mixed with 500 µl phenol/choloroform solution

ChIP sequencing library preparation and sequencing

The construction of library was performed using NEBNext® Ultra™ DNA Library Prep Kit for Illumina

(New England Biolabs, UK) according to the manufacturer's instructions. Briefly, the chipped DNA was ligated with adaptors. After purification, PCR reaction was done with adaptor-ligated DNA and index primer for multiplexing sequencing. Library was purified by using magnetic beads to remove all reaction components. The size of library was assessed by Agilent 2100 bioanalyzer (Agilent Technologies, Amstelveen, The Netherlands). High-throughput sequencing was performed as paired-end sequencing (101 bp) using HiSeq 2500 (Illumina, Inc., USA).

ChIP sequencing data analysis

The reads were trimmed and aligned using Bowtie2 (Langmead and Salzberg, 2012). Bowtie2 indices were generated from genome assembly sequence or the representative transcript sequences during the alignment of the reads to the genome or transcriptome, respectively. With the mapped reads, we used MACS2 (Model-base Analysis of ChIP-seq) for identifying the peaks representing DNA binding of KDM5A. Transcription factor binding sites enriched in the peaks were identified using HOMER (Hypergeometric Optimization of Motif EnRichment), a software suite for ChIp-Seq analysis. Gene classification was performed based on the results from functional enrichment analysis using g:Profiler (https://biit.cs.ut.ee/gprofiler/).

Protein extraction

Collected hippocampal tissues from two control (LV-GFP) and two Aβ (LV-Mito-Aβ) groups were individually pulverized using Covaris CP02 Cryoprep device (Covaris, Woburn, MA). Briefly, each tissue piece was placed in a Covaris tissue bag (Covaris, 430487), and the bag was placed in a liquid nitrogen for 30 s. The frozen tissue was subsequently pulverized at impact level 2. We then added 500 μl of lysis buffer (4 % SDS in Tris-HCl pH 7.6 and phosphatase inhibitor) to the tissue powder, which were subsequently sonicated using a probe sonicator (Q55, QSONICA, Newtown, CT) at 30 W on ice for 30 s. The tissue lysate was centrifuged at 16,000 g for 10 min, and supernatants was transferred to

a siliconized low-retention tube. Protein concentration was then measured using the BCA protein assay (Pierce, Rockford, IL).

Protein digestion

All hippocampal proteins were digested by the filter aided sample preparation (FASP) method[1] with a slight modification. Briefly, hippocampal proteins were reduced with SDT buffer (4 % SDS in 0.1 M Tris-HCl pH 7.6 and 0.1 M DTT) at 37 °C for 45 min by shaking at 300 rpm followed by boiling for 10 min at 95 °C on a thermomixer (Eppendorf, Hamburg, Germany). The protein sample was centrifuged at 16,000 g for 5 min and transferred to a YM-30 microcon filter, in which the protein sample was mixed with 200 µl of UA buffer (8 M urea in 0.1 M Tris-HCl pH 8.5). The protein samples were then centrifuged at 14,000 g at 20 °C for 60 min to remove SDS. This step was repeated three times. After alkylation for 25 min with 100 µL of 0.05 M iodoacetamide in 8 M urea at room temperature in the dark, the filter was centrifuged at 14,000 g for 30 min, followed by washing with 200 µl of UA buffer four times. 100 µL of 0.05 M ammonium bicarbonate was added to the filter before it was centrifuged at 14,000 g for 30 min for buffer exchange. This step of buffer exchange was repeated twice. Subsequently, sequencing grade modified trypsin (Promega; Madison; WI) was added to protein samples on the filter at an enzyme to protein ratio of 1:50 (w/w), and the proteins were digested overnight at 37 °C. After the first digestion, the second digestion was carried out with trypsin (1:100 w/w) at 37 °C for 6 hrs. The concentration of hippocampal peptide samples was measured by the BCA assay.

Isobaric Tag for Relative and Absolute Quantitation (iTRAQ) labeling and fractionation

The hippocampal peptide samples from two LV-GFP and two LV-mitoAβ injected groups (700 µg each) were labeled with 4-plex iTRAQ reagent (AB Sciex, Foster City, CA) according to the manufacturer's instructions. The two LV-GFP groups were labeled with 114/116 iTRAQ reagents, respectively, while

two LV-mitoAβ groups were labeled with 115/117 iTRAQ reagents, respectively. All labeled peptide samples were then pooled and dried by vacuum centrifugation. The pooled iTRAQ labeled peptide sample was fractionated using Agilent 1269 Infinity HPLC system (Agilent, Palo Alto, CA) as previously described [2]. The Xbridge C18 column (4.6 mm × 250 mm, 130 Å, 5 μm, Waters, Milford, MA) and a guard column (4.6 mm × 20 mm, 130 Å, 5 μm) were used for the peptide separation. The peptide fractionation was performed with a 115 min gradient at a flow rate of 500 μL/min: at 0 % solvent B [10 mM TEAB in 90 % ACN (pH 7.5)] for 10 min, from 0 % - 5 % solvent B in 10 min, from 5 % to 35 % in 60 min, from 35 % - 70 % in 15 min, at 70 % solvent B for 10 min, from 70 % - 0 % solvent B in 10 min, and at 0 % solvent over 15 min. A total of 96 fractions were collected in every 1 min from 15 min to 110 min, and they were non-contiguously concatenated into 24 fractions (i.e., #1-#25-#49-#73, #2-#26-#50-#74, …, #24-#48-#72-#96). The resultant 24 fractions were dried by vacuum centrifugation. 10 μg of each fraction was used for global proteome profiling, and the remaining samples were used for phosphopeptide enrichment by immobilized-metal affinity chromatography (IMAC) [2]. For IMAC experiment, the 24 fractions were further concatenated into 12 fractions by pooling two adjacent fractions (i.e., #1-#2, #3-#4, …, #23-#24) before drying and storing at -80 °C.

Phosphopeptide enrichment

For phosphopeptide enrichment, 1.5 mL of Ni-NTA bead slurry (36113, Qiagen, Valencia, CA) was washed three times with 1.2 mL of deionized water (DIW), and Ni^{2+} ions were removed from NTA bead by adding 1.2 mL of 100 mM EDTA (pH 8.0) and mixing for 30 min on an end-over-end rotator (SB3, Stuart). After removal of the EDTA solution, NTA beads were washed three times with 1.2 mL of DIW and then reacted with 1.2 mL of 10 mM aqueous $FeCl_3$ solution for 30 min on an end-over-end rotation. The Fe^{3+}-NTA beads were washed with 1.2 mL of DIW three times and resuspended in 1.2 mL of 1:1:1 ACN/MeOH/0.01 % acetic acid for aliquoting into 12 microcentrifuge tubes and each of the Fe^{3+}-NTA beads was washed with 400 μL of binding buffer (80 % ACN/0.1% TFA). Each of the 12 fraction samples was then resuspended in 500 μL of binding buffer and individually transferred to a tube of the

aliquoted Fe^{3+}-NTA beads. The binding reactions of the 12 aliquoted peptide samples were performed in parallel for 30 min on an end-over-end rotation. After the binding reaction, the beads were washed with 500 µL of binding buffer for four times. The bound phosphopeptides were eluted by incubating in 125 µL of 1:1 ACN/2.5 % ammonia in 2 mM phosphate buffer (pH 10) for 1.5 min. The eluted phosphopeptides were acidified with 10 % TFA to pH 3.5 - 4.0 immediately before drying.

LC-MS/MS experiments

The 24 fraction global peptide and 12 fraction phosphopeptide samples from hippocampal tissues were individually analyzed using a proteome profiling platform (at the Center for Proteogenomic Research, kore) that consists of a dual-online UPLC system (Waters, Milford, MA) [3] online coupled with Q-Exactive orbitrap mass spectrometer (ThermoScientific, Bremen, Germany). The dual-online UPLC system was equipped with two analytical columns (75 µm i.d. x 360 µm o.d, 100 cm) and two solid phase extraction (SPE) columns (150 µm i.d. x 360 µm o.d, 3 cm), and all columns were manufactured by slurry packing of C18 materials (3 µm diameter, 300 Å pore size, Jupiter, Phenomenex, Torrance, CA, USA) as previously reported[4]. The column was heated to 60 °C, and a flow rate was set to 300 nL/min[4]. The global peptides were separated by a 180 min linear gradient (1-40 % solvent B over 160 min, 40 - 80 % over 5 min, 80 % for 10 min and holding at 1 % for 5 min), and phosphopeptides were separated by a 240 min gradient (1 % to 50 % solvent B over 220 min, 50 % - 80 % solvent B over 5 min, 80 % solvent B for 10 min and holding at 1 % for 5 min). The solvent A and B were 0.1 % formic acid in water and 0.1 % formic acid in acetonitrile, respectively. The eluted peptides from LC were ionized at 2.4kV, and the desolvation capillary temperature was kept at 250 °C. Full MS data was acquired between 400 and 2,000 m/z at the resolution of 70,000, and the automated gain control (AGC) target value was set to 1.0×10^6 with a maximum ion injection time of 20 ms. The tandem mass (MS/MS) data were obtained using a data-dependent acquisition (DDA) mode, and isolation of the most abundant top 10 ions was performed within ± 0.8 Th window. The isolated precursor ion was fragmented at a normalized collision energy (NCE) of 30 for higher energy collisional dissociation (HCD). The

resolution of MS/MS data was set to 17,500, and AGC target value was to 1.0 x 10^6 with a maximum ion injection time of 60 ms.

LC-MS/MS data analysis

The LC-MS/MS data from global proteome and phosphoproteome profiling experiments were processed with the PE-MMR (Post-experiment monoisotopic mass refinement) method as previously described[5]. The resultant tandem mass data were searched against a mouse uniprot database (April, 2014; 51,388 entries) through MS-GF+ (v9387) search engine[6] with the search parameters: Semi tryptic, 10 ppm precursor mass tolerance, iTRAQ (+144.102063 Da) modification of lysine and N-termini and carbamidomethylation (+57.0214 Da) of cysteine as static modifications; and methionine oxidation (+15.994915 Da) as a variable modification. For phosphopeptide search, phosphorylation (+79.966) to serine/threonine/tyrosine was set as an additional variable modification. The identified phosphopeptides were further subjected to the unique mass class (UMC) filtering to localize the sites of phosphorylation as previously described[7] . The search results from the 24 MS/MS data sets of global proteome and the 12 MS/MS data sets from phosphoproteome were combined, respectively. Peptides were finally identified from the peptide spectrum matches (PSMs) with the false discovery rate (FDR) less than 0.01 and used for the downstream analyses. The identified non-redundant peptides were used to obtain protein groups by a bipartite graph analysis[8]. Among the component proteins of each protein group, the protein of the highest number of associated peptides was selected as the representative of the protein group. When multiple proteins had the same number of non-redundant peptides, the protein of a higher protein sequence coverage was chosen as the representative protein. Additionally, a protein having any unique peptides was selected as a separate representative protein. For global proteome analysis, two or more sibling non-redundant peptides were required for protein group identification. In the case of the phosphoproteome analysis, all protein groups identified by the phosphorylated peptides were used.

Identification of differentially expressed peptides and proteins

The intensities of the iTRAQ reporter ions for hippocampus samples from two LV-GFP and two LV-Mito-Aβ injected mice were normalized using the quantile normalization method[9]. Using the normalized intensities of each peptide, we performed Student t-test and calculated log_2-median-ratios (fold-changes) for LV-Mito-Aβ to LV-GFP groups. We next estimated the null hypothesis distributions of these values by performing random permutations of the samples and computing t-statistic values and log_2-median-ratios as previously described[10]. An adjusted p value was calculated for a t-statistic value for each peptide by two sample test using the empirical distribution. Differentially expressed (DEpeptides) and phosphorylated peptides (DPpeptides) were then identified as the peptides with $p < 0.05$ from the two sample t-test and log_2-median-ratios > 95th percentiles in its empirical null distribution (1.32- and 1.58-fold for global and phosphoproteome profiles, respectively). The DEPs were identified as the proteins with at least two unique DEpeptides showing consistent up- or down-regulation in LV-mitoAβ injected mice. DPpeptides with more than two spectral counts were used for the subsequent analyses, and differentially phosphorylated proteins (DPPs) were defined as the proteins containing the DPpeptides.

GOBP association analysis.

We built a network model describing the links among the 586 GOBPs enriched by the three sets of proteins affected by LV-mitoAβ: 1) 281 up- and 2) 218 down-regulated proteins and 3) 191 DPPs. For each pair of the 586 GOBPs (GOBP1 with n proteins, GOBP2 with m proteins, and k shared proteins between GOBP1 and 2), we computed the similarity score as $2k/(n+m)$ and connected the two GOBPs with the similarity score > 0.52 (99 % of the null distribution for the similarity score). This procedure resulted in 43 connected subnetworks, called functional modules. The importance (weighted degree) of GOBPs in each module was estimated by summing the similarity scores of its interactors in the corresponding subnetwork. The GOBPs were then ranked by their weighted degrees such that the top-ranked GOBP has the largest weighted degree (i.e., a hub-like term). Also, the key GOBPs should be highly enriched with up- and down-regulated proteins and DPPs. Thus, we evaluated the enrichment of the GOBPs in each module using EASE scores obtained from DAVID[11]. The GOBPs in each module

was then ranked such that the top-ranked GOBP has the largest enrichment score. After summing the two ranks from the weighted degrees and the enrichment scores, finally, the key GOBP in each module was selected as the top-ranked one based on the summed ranks.

Supplementary Table 1. List of Primers Used to Perform RT-PCR.

**Gene**	**Primer sequences**	
	Forward	Reverse
KDM5A	TGAACGATGGGAAGAAAAGG	AGCGTAATTGCTGCCACTCT
BDNF	GCTGCAGAACAGAAGGAGTACA	GTCCTCATCCAACAGCTCTTCTATC
MEF2A	GTGTACTCAGCAATGCCGAC	AACCCTGAGATAACTGCCCTC
SOX2	TCCCCCCTTTTATTTTCCGTAG	CCTGATTCCAATAACAGAGCCG
MSL1	CCGGAGTTACACAGGCCTTG	GGGATAGCTGTGAGCTCGGG
TUJ1	ACCTCAACCACCTGGTATCG	GGGTACCACTCCACGAAGTA
18s rRNA	GTAACCCGTTGAACCCCATT	CCATCCAATCGGTAGTAGCG

Supplementary Figures

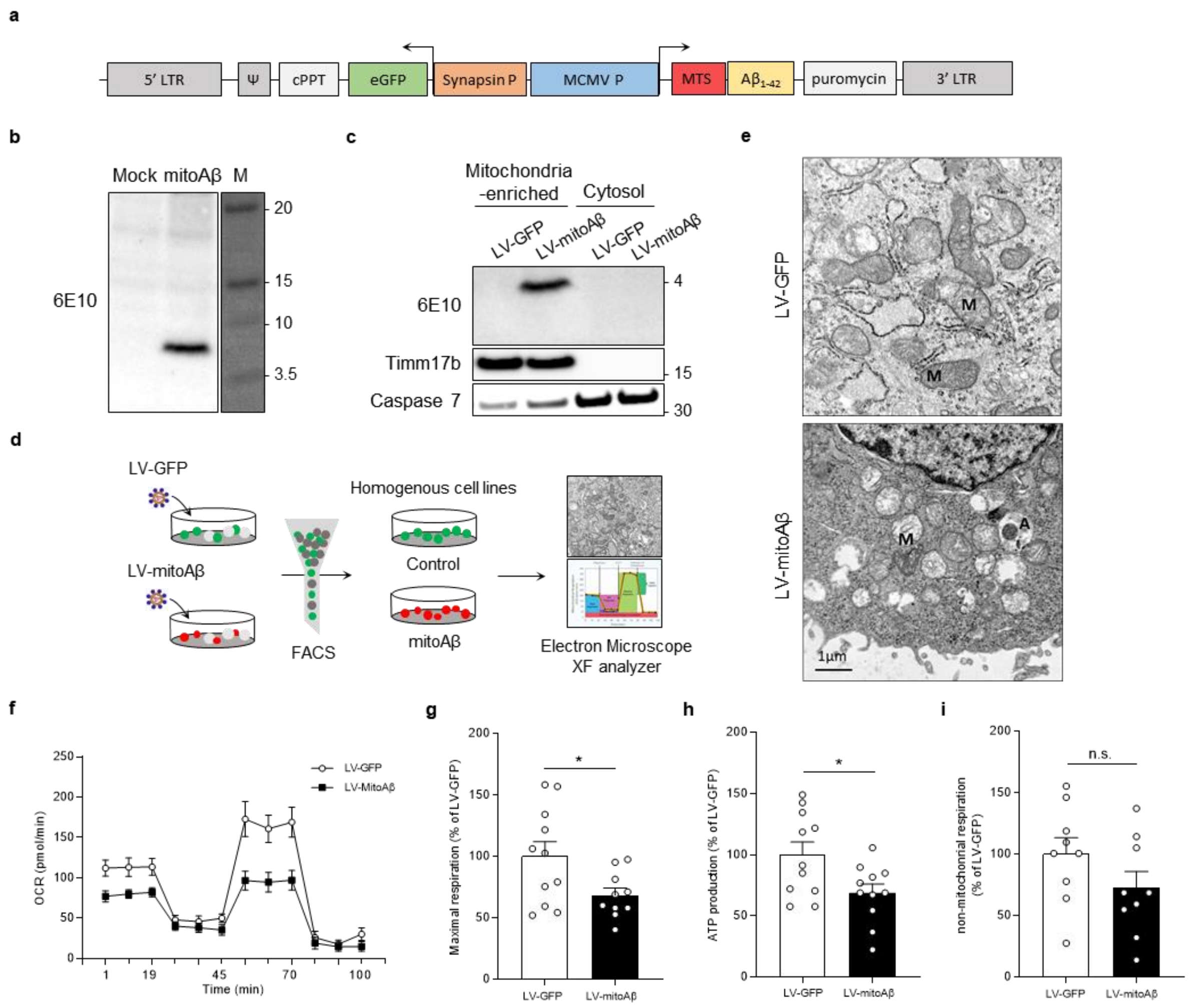

Supplementary Fig. 1. Mitochondria-targeted Aβ disrupts mitochondrial morphology and function. a Diagram of mitoAβ DNA construct used in the study. **b** The expression of transfected mitoAβ in CHO cells, confirmed by the western blot analysis. **c** Subcellular localization of mitoAβ transduced by a lentiviral system was confirmed by the mitochondria-enriched fractionation. Timm17b was used as a marker for mitochondria-enriched samples and caspase 7 was used as a marker for the cytoplasm. **d** Experimental procedure of making homogenous cells expressing mitoAβ. Before analyzing cellular respiration and transmission electron microscope imaging, mitoAβ was transduced into the HT22 cell line using a lentiviral system, and GFP^{+} or GFP/mitoAβ^{+} cells were collected by FACS. **e** Mitochondria in the cytoplasm of GFP^{+} or GFP/mitoAβ^{+} cells imaged by transmission electron microscopy. M: Mitochondria, A: Autophagosome. **f** Cellular respiration rate was measured by an XF

analyzer. **g-i** Quantitative analysis of the maximum respiratory rate, ATP production amount, and mitochondria-independent respiratory rates of GFP^+ or GFP/mito$A\beta^+$ cells. Unpaired t-test, $n = 9 - 11$ in each group. All results are represented as mean ± SEM. * $P < 0.05$.

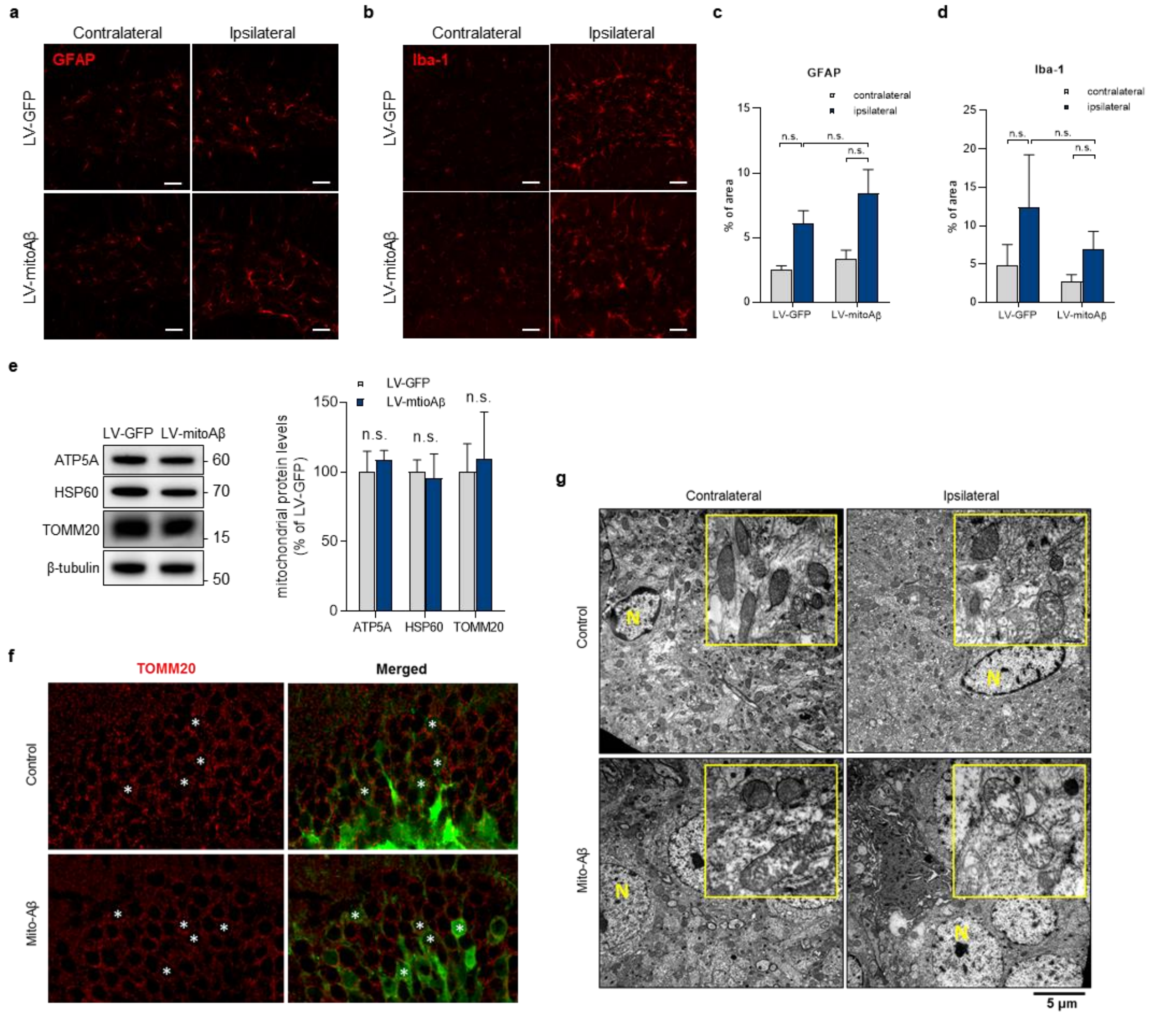

Supplementary Fig. 2. The characteristics of mitoAβ expression in the hippocampus. a-d Representative images showing glial activation, such as GFAP (**a** and **c**) and Iba-1 (**b** and **d**), of contralateral and ipsilateral hippocampus after stereotaxic injection of lentivirus. Stereotaxic injection did not significantly increase, but caused an inflammatory response with increasing tendency, but mitoAβ expression did not specifically affect neuroinflammation in the hippocampus. Scale bar, 30 μm. Two-way ANOVA analysis, n = 6 – 8 mice in each group. **e** MitoAβ expression did not alter mean value of LV-GFP group for each protein. Unpaired t-test, n = 4 mice in each group. **f** Representative images showing immunostaining of mitochondrial protein, TOMM20 in GFP^{+} or GFP/mitoAβ^{+} cells of the hippocampus. g Representative images showing mitochondrial morphology

in the hippocampus of LV-GFP or LV-mitoAβ group using TEM. N: Nucleus. Scale bar, 5 μm. All results are represented as mean ± SEM.

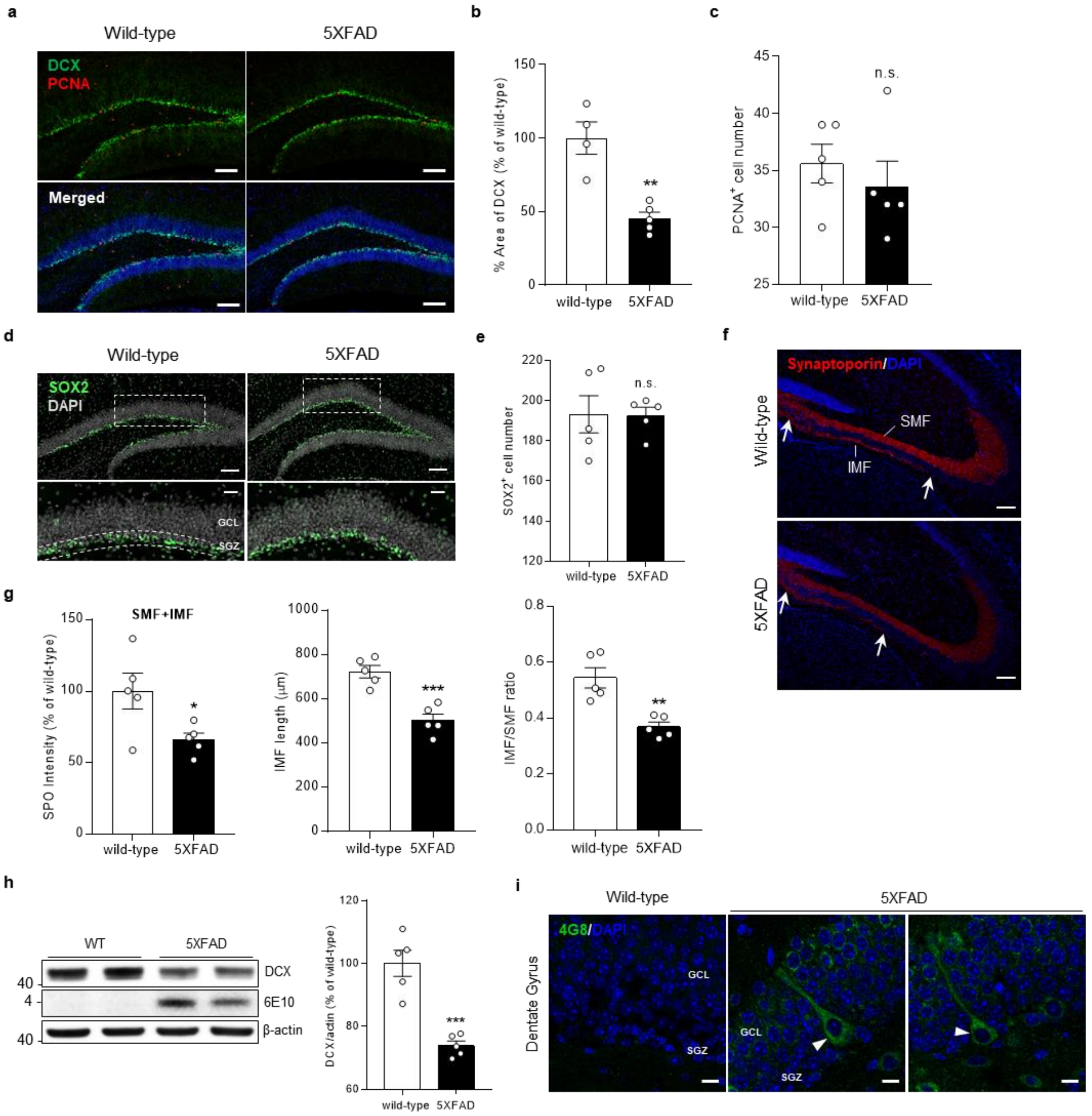

Supplementary Fig. 3. Inhibition of neuronal differentiation and AHN deficits in 6-week-old 5XFAD mice. a Immunostaining of DCX and PCNA in the DG of wild-type and 5XFAD mice. Scale bar, 100 µm. **b** Quantitative analysis of DCX intensities in the DG of wild-type and 5XFAD mice. Unpaired t-test, *n* = 4 (wild-type) or 5 (5XFAD) mice. **c** Quantitative analysis of the number of PCNA^{+} cells in the DG of wild-type and 5XFAD mice. Unpaired t-test, *n* = 5 mice in each group. **d** Immunostaining of SOX2 in the DG of wild-type and 5XFAD mice. Scale bar, 100 µm. The enlarged image showing SOX2 staining in the SGZ. Scale bar, 100 µm. **e** Quantitative analysis of the number of SOX2^{+} cells in the SGZ of wild-type and 5XFAD mice. Unpaired t-test, *n* = 5 mice in each group. **f**

Immunostaining of synaptoporin in the hippocampus of wild-type and 5XFAD mice. Arrows indicates the beginning and end of the IMF. SMF: superficial mossy fiber, IMF: Infrapyramidal mossy fiber. Scale bar, 100 μm. **g** Quantitative analysis of synaptoporin intensities for both SMF and IMF, IMF length, and IMF/SMF ratio in wild-type and 5XFAD mice. Unpaired t-test, $n = 5$ mice in each group. **h** Reduced DCX levels and presence of Aβ confirmed by western blot analysis in the hippocampus of wild-type and 5XFAD mice. Unpaired t-test, $n = 5$ mice in each group. **i** A representative image showing the presence of Aβ in the SGZ of wild-type and 5XFAD mice. Arrow heads indicate intraneuronal accumulation of Aβ in granule cells. SGZ: Subgranule zone, GCL: Granule cell layer. Scale bar, 10 μm. All results are represented as mean ± SEM. * $P < 0.05$; ** $P < 0.01$; *** $P < 0.001$.

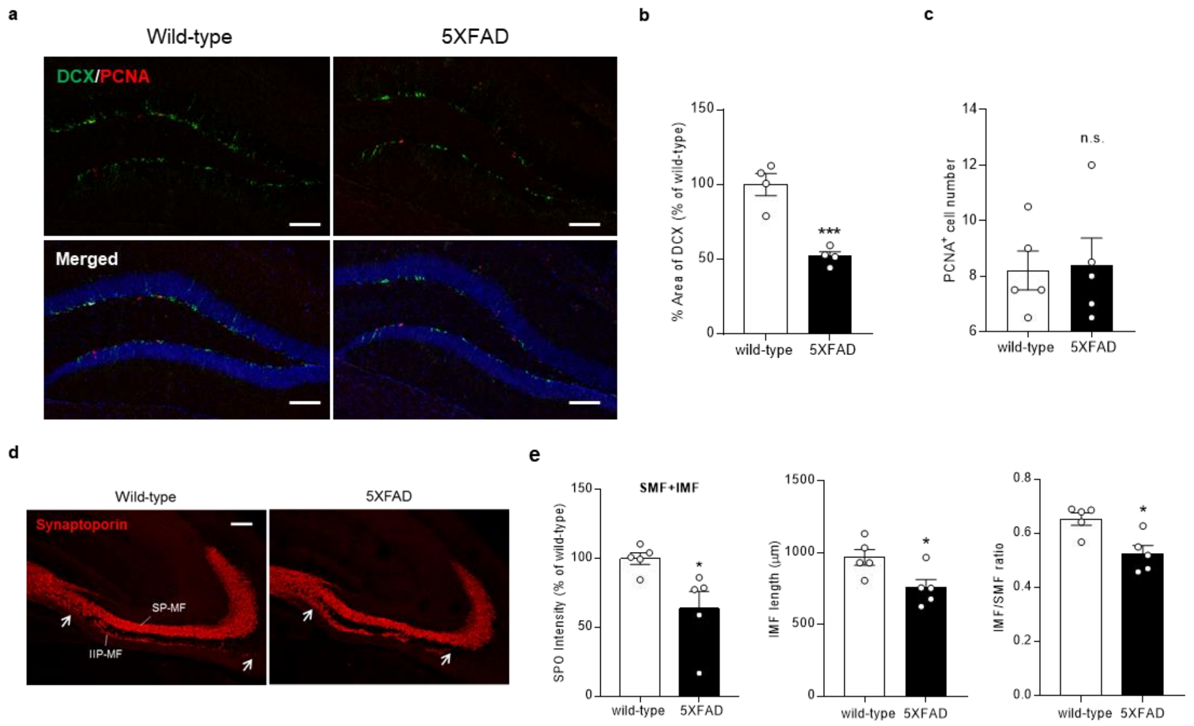

Supplementary Fig. 4. Inhibition of neuronal differentiation and AHN deficits in 20-week-old 5XFAD mice. a Immunostaining of DCX and PCNA in DG of 20-week-old wild-type and 5XFAD mice. Scale bar, 100 μm. **b** Quantitative analysis of DCX intensities in DG of 20-week-old wild-type and 5XFAD mice. Unpaired t-test, $n = 4$ mice in each group. **c** Quantitative analysis of the number of PCNA^{+} cells in DG of 20-week-old wild-type and 5XFAD mice. Unpaired t-test, $n = 5$ mice in each group. **d** Immunostaining of synaptoporin in the hippocampus of 20-week-old wild-type and 5XFAD mice. Arrows indicates the beginning and end of the IMF. SMF: superficial mossy fiber, IMF: Infrapyramidal mossy fiber. Scale bar, 100 μm. **e** Quantitative analysis of synaptoporin intensities for both SMF and IMF, IMF length, and IMF/SMF ratio in 20-week-old wild-type and 5XFAD mice. Unpaired t-test, $n = 5$ mice in each group. All results are represented as mean ± SEM. * $P < 0.05$; *** $P < 0.001$.

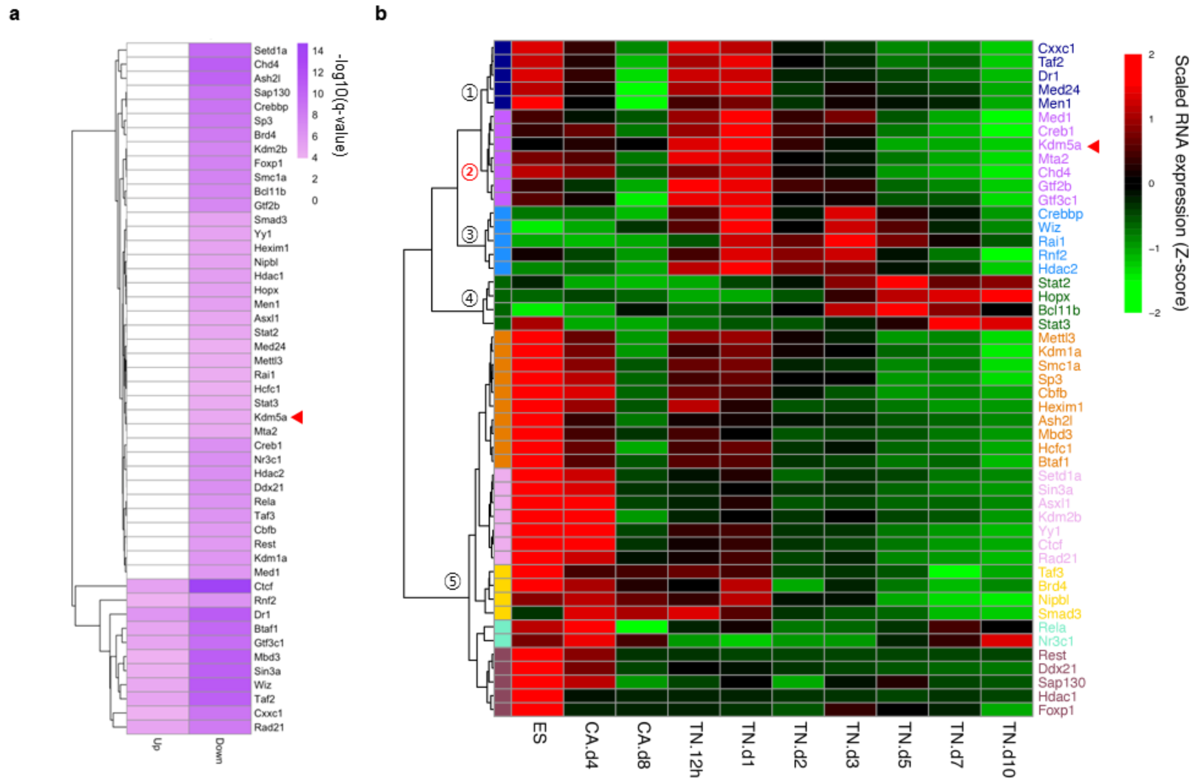

Supplementary Fig. 5. Search for putative upstream-binding factors controlling hippocampal proteome changes by mitoAβ expression. a Heat map depicting 49 upstream regulators predicted to bind to genes that encode 281 up-regulated and 218 down-regulated DEPs (total 499 DEPs) in hippocampal proteome (**Fig. 2a**). **b** Hierarchical clustering heat map of RNA expression pattern for 49 upstream regulators (**a**) along the in vitro neurogenesis. Hierarchical clustering groups upstream regulators into 5 clusters: (1) ES/early differentiation, (2) early differentiation, (3) mid differentiation, (4) late differentiation, and (5) ES cell.

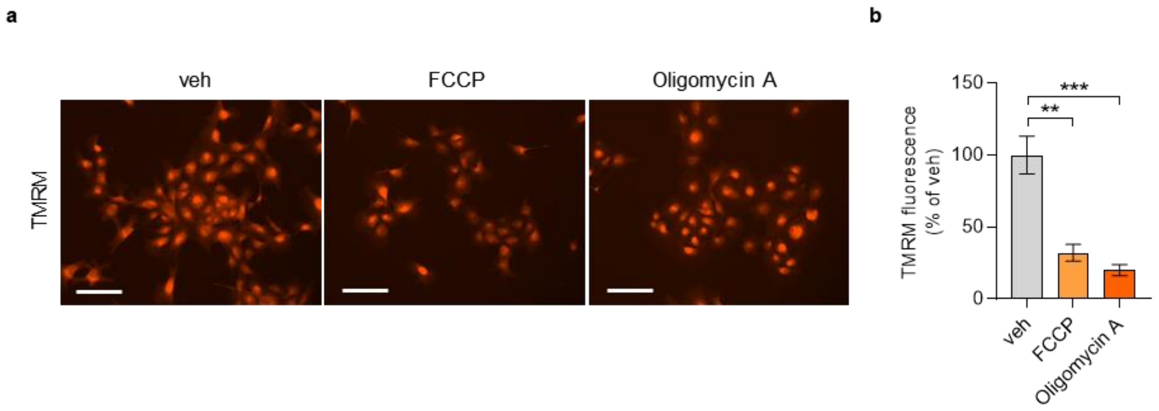

Supplementary Fig. 6. Loss of mitochondrial membrane potential in neural progenitors by drug-induced mitochondrial toxicity. a Representative images of TMRM-stained mitochondrial membrane potential of neural progenitors after FCCP (3 μM, 12 hrs) or oligomycin A (3 μM, 12 hrs) treatment. Scale bar, 100 μm. **b** Quantitative analysis of TMRM fluorescence in neural progenitors. One-way ANOVA, $n = 3 – 5$. Results are represented as mean ± SEM. ** $P < 0.01$; *** $P < 0.001$.

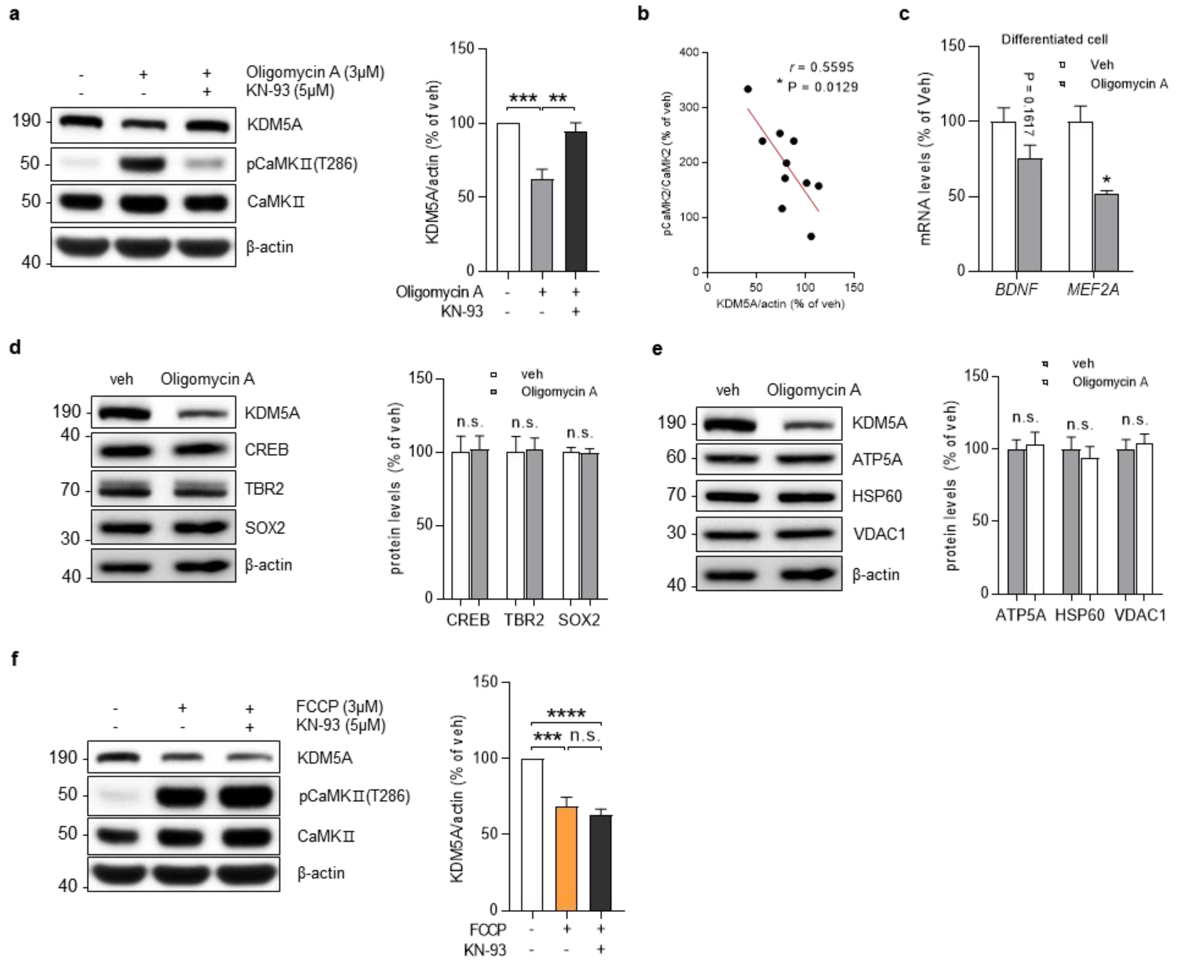

Supplementary Fig. 7. The effects of oligomycin A on KDM5A degradation and neuronal differentiation. a The decrease of KDM5A by oligomycin A (3 μM for 12 hrs) was restored by the treatment with a CaMKII inhibitor, KN-93. Data was normalized to β-actin. One way-ANOVA analysis, *n* = 6 in each group. **b** Correlation analysis between phosphorylation of CaMKII and KDM5A abundance in neural progenitors treated with oligomycin A (3 μM for 12 hrs). Linear regression analysis, *n* = 10 in total. **c** After the differentiation of neural progenitors treated with FCCP or oligomycin A (3 μM for 12 hrs), *BDNF* and *MEF2A* mRNA levels were measured in differentiated cells (5 days) by RT-PCR. **d** The expression levels of transcription factors required for the differentiation in neural progenitors treated with oligomycin A (3 μM for 12 hrs). Data was normalized to β-actin. Unpaired t-test for each protein, *n* = 4 in each group. **e** The expression levels of mitochondrial proteins in ReN cells treated with oligomycin A (3μM for 12 hrs) Data was normalized to β-actin. Unpaired t-test for each protein, *n* = 4 in each group. **f** KDM5A protein levels, which decrease as a result of FCCP treatment (3

μM for 12 hrs), were not recovered by KN-93. Data was normalized to β-actin. One way-ANOVA analysis, $n = 6$ in each group. All results are represented as mean ± SEM. * $P < 0.05$; ** $P < 0.01$; *** $P < 0.001$; **** $P < 0.0001$.

References

1 Wisniewski, J. R., Zougman, A., Nagaraj, N. & Mann, M. Universal sample preparation method for proteome analysis. *Nat. Methods.* **6**, 359-362 (2009).

2 Park, J. M. *et al.* Integrated analysis of global proteome, phosphoproteome, and glycoproteome enables complementary interpretation of disease-related protein networks. *Sci. Rep.* **5**, 18189 (2015).

3 Lee, H. *et al.* A simple dual online ultra-high pressure liquid chromatography system (sDO-UHPLC) for high throughput proteome analysis. *Analyst* **140**, 5700-5706 (2015).

4 Hyung, S. W., Kim, M. S., Mun, D. G., Lee, H. & Lee, S. W. The effect and potential of using a temperature controlled separation column with ultra-high pressure microcapillary liquid chromatography/tandem mass spectrometry on proteomic analysis. *Analyst* **136**, 2100-2105 (2011).

5 Shin, B. *et al.* Postexperiment monoisotopic mass filtering and refinement (PE-MMR) of tandem mass spectrometric data increases accuracy of peptide identification in LC/MS/MS. *Mol. Cell. Proteomics* **7**, 1124-1134 (2008).

6 Kim, S. *et al.* The generating function of CID, ETD, and CID/ETD pairs of tandem mass spectra: applications to database search. *Mol. Cell. Proteomics.* **9**, 2840-2852 (2010).

7 Madar, I. H. *et al.* Reduction of Ambiguity in Phosphorylation-site Localization in Large-scale Phosphopeptide Profiling by Data Filter using Unique Mass Class Information. *B. Korean. Chem. Soc.* **35**, 845-850 (2014).

8 Zhang, B., Chambers, M. C. & Tabb, D. L. Proteomic parsimony through bipartite graph analysis improves accuracy and transparency. *J. Proteome. Res.* **6**, 3549-3557 (2007).

9 Bolstad, B. M., Irizarry, R. A., Astrand, M. & Speed, T. P. A comparison of normalization methods for high density oligonucleotide array data based on variance and bias. *Bioinformatics* **19**, 185-193 (2003).

10 Chae, S. *et al.* A systems approach for decoding mitochondrial retrograde signaling pathways. *Sci. Signal.* **6**, rs4 (2013).

11 Huang da, W., Sherman, B. T. & Lempicki, R. A. Systematic and integrative analysis of large gene lists using DAVID bioinformatics resources. *Nat. Protoc.* **4**, 44-57 (2009).
